# Weight gain among children under five with severe malnutrition in therapeutic feeding programmes: a systematic review and meta-analysis

**DOI:** 10.1016/j.eclinm.2025.103083

**Published:** 2025-02-12

**Authors:** Grace O'Donovan, Daniel Allen, Thandile Nkosi-Gondwe, Kenneth Anujuo, Mubarek Abera, Amir Kirolos, Laurentya Olga, Debbie Thompson, Kimberley McKenzie, Elizabeth Wimborne, Tim J. Cole, Albert Koulman, Natasha Lelijveld, Amelia C. Crampin, CHANGE Study Collaborators Group, Grace O'Donovan, Daniel Allen, Thandile Nkosi-Gondwe, Kenneth Anujuo, Mubarek Abera, Amir Kirolos, Laurentya Olga, Debbie Thompson, Kimberley McKenzie, Elizabeth Wimborne, Tim J. Cole, Albert Koulman, Natasha Lelijveld, Amelia C. Crampin, Suvi T. Kangas, Gemechu Ameya, Asha Badaloo, Charles Opondo, Marko Kerac, Charles Opondo, Marko Kerac

**Affiliations:** a Department of Population Health, London School of Hygiene and Tropical Medicine, London, UK; b Malawi Epidemiology and Intervention Research Unit (MEIRU), Malawi; c Medical Research Council, Epidemiology Unit, School of Clinical Medicine, University of Cambridge, Cambridge, UK; d Department of Psychiatry, Faculty of Medical Science, Jimma University, Jimma, Ethiopia; e Department of Women and Children's Health, Institute of Life Course and Medical Sciences, University of Liverpool, Liverpool, UK; f Core Metabolomics and Lipidomics Laboratory, Institute of Metabolic Science, University of Cambridge, Cambridge, UK; g Caribbean Institute for Health Research, University of the West Indies, Kingston, Jamaica; h School of Human Development and Health, Faculty of Medicine, University of Southampton, Southampton, UK; i UCL Great Ormond Street Institute of Child Health, London, UK; j Emergency Nutrition Network, Kidlington, Oxfordshire, UK; k Department of Medical Statistics, London School of Hygiene and Tropical Medicine, London, UK

**Keywords:** Severe malnutrition, Weight gain, Therapeutic feeding programmes, Child health, Catch-up growth

## Abstract

**Background:**

Globally, some 45 million children under five years of age are wasted (low weight-for-height). Although 2023 World Health Organisation guidelines on their care did not aim to identify optimal weight gain, they did mention 5–10 g/kg/day as a target, which is a change from prior guidelines that recommended 10–15 g/kg/day, when inpatient-only care was the norm. We aimed to inform future policy/programming on weight gain targets.

**Methods:**

For this systematic review and meta-analysis, we searched Embase, Global Health and Medline. The final search was on 23/02/2024. Papers were included if they reported weight gain of children aged 6–59 months with severe malnutrition during inpatient (facility-based), outpatient (home-based), and hybrid treatment (initially inpatient and progressing to outpatient treatment). Summary data were extracted, and quality was assessed using a NICE Quality Appraisal Checklist. Our primary outcome was mean rate of weight gain (g/kg/day) during treatment. We conducted random-effects meta-analysis to describe pooled mean weight gain by programme type. Meta-regression investigated potential associations of weight gain with length of stay and programme outcomes. We registered the study on PROSPERO (CRD42023266472).

**Findings:**

Our search yielded 3173 papers. We reviewed 321 full texts, identifying 126 eligible papers. Of these, 104 papers, including some 240,650 participants, reported weight gain as g/kg/day and were eligible for meta-analysis. Mean rate of weight gain was 8.8 g/kg/day (95% CI: 7.6, 9.9; I^2^ = 97.8%) across 18 inpatient programmes, 3.4 g/kg/day (95% CI: 2.0, 4.7; I^2^ = 99.4%) across 12 hybrid programmes, and 3.9 g/kg/day (95% CI: 3.4, 4.4; I^2^ = 99.7%) across 60 outpatient programmes. We found inconsistent evidence of an association between slower weight gain and higher mortality: there was weak evidence of association after adjusting for programme type (coefficient = −0.4; 95% CI: −0.7, −0.02; p = 0.04; n = 118 programmes). There was high heterogeneity between studies. Details of weight gain calculation methods varied. We found no evidence for publication bias when accounting for programme type (Egger's test p-value = 0.2).

**Interpretation:**

Weight gain in outpatient programmes was markedly slower than in inpatient treatment. Clearer reporting of weight gain and a better understanding of the sequelae of faster/slower recovery is important to set future weight gain targets. Our results set an important baseline for current programmes to benchmark against.

**Funding:**

Medical Research Council/Global Challenges Research Fund, grant number: MR/V000802/1.

**Research in context**

**Evidence before this study**

Globally, 45 million children under five are wasted. Treatment focus has shifted from inpatient care towards outpatient-based programmes, particularly Community-based Management of Acute Malnutrition (CMAM).

Before this study, we searched the literature for previous studies reporting weight gain in children under five during treatment for severe malnutrition. Weight recovery in outpatient care was generally slower than prior inpatient-based targets of 10–15 g/kg/day. There was some concern that too slow a weight gain reflects poor recovery. Balancing this was evidence that too rapid weight catch-up may be associated with cardiometabolic risk later in life. We found previous systematic reviews that considered weight gain of children with severe malnutrition by treatment programme type but only as one of many outcomes, and without meta-analysis or with <5 studies in the meta-analysis.

**Added value of this study**

As our systematic review is the first to focus on weight gain during treatment for severe childhood malnutrition by programme type, we call attention to this important consideration. Our findings indicate that average weight gain in outpatient programmes is slower compared to inpatient programmes, and that average outpatient weight gain is below current World Health Organisation targets.

We found inconsistent evidence for a weak association between weight gain and mortality, after adjusting for programme type. Slow programme-level weight gain may be a red flag for a high burden of more severely wasted or sick children, or may reflect more adverse community conditions or programmatic quality issues.

**Implications of all the available evidence**

While the treatment of severe childhood malnutrition is of major public health importance, the optimal weight gain remains unknown. Our novel meta-analysis focussing on average weight gain by programme type forms an important benchmark for evaluation of existing programmes.

Our findings, accompanied with previous research, highlight the need for more evidence to identify optimal weight gain, balancing the short-term and potential long-term effects. Towards this, guidelines for the clear reporting of weight gain and calculation methods would help improve consistency.

## Introduction

Globally, in 2022, some 45 million children aged under five years were wasted (low weight-for-height),^1^ a serious form of malnutrition which accounts for 875,000 deaths in children under five each year.^2^ Severe malnutrition is “any form of malnutrition (undernutrition) associated with high risk of severe adverse outcomes”.^3^ Severely low weight-for-height, severely low mid-upper arm circumference (MUAC) and/or oedematous malnutrition (kwashiorkor) are known as types of “Severe Acute Malnutrition” (SAM). Criteria defining SAM have changed over time.^4^ Current recommendations are that SAM in children aged 6–59 months is identified as weight-for-height z-score (WHZ) <−3 of the World Health Organisation (WHO) Child Growth Standards, MUAC <115 mm, and/or bilateral oedema.^4^

Historically, children with SAM were treated in inpatient facilities using a clinically-focused model of care with specially-formulated therapeutic milks.^4^, ^5^, ^6^ Over the last 20 years, this has shifted to a public health model of “Community-Management of Acute Malnutrition” (CMAM) programmes, to improve coverage and achieve low case-fatality rates.^7^ In CMAM, children with uncomplicated SAM (i.e., with appetite and no medical complications), which is usually >80%, are treated as outpatients in the community using Ready-to-Use Therapeutic Foods (RUTF).^7^^,^^8^ Children with complicated SAM (i.e., poor appetite and/or medical complications) and those who failed to gain weight as outpatients are treated as inpatients. Recent years have also seen the rise of simplified and combined programmes, which respectively seek to simplify admission criteria and treatment protocols, and integrate treatment of severe and moderate wasting.

This review arises from observations by the study team that rates of weight gain in outpatient-based therapeutic feeding programmes, including outpatient care in CMAM, are often markedly slower than in traditional inpatient programmes. Guidelines from 1999 described a “usual” weight gain of 10–15 g/kg/day (g/kg/day), with <5 g/kg/day triggering concern about poor recovery and high mortality risk.^6^ The emphasis on rapid recovery (quantified by weight gain) is contrasted with more recent evidence suggesting that rapid post-malnutrition weight gain is associated with adverse long-term cardiometabolic risk in survivors.^9^, ^10^, ^11^ While previous reviews have investigated weight gain by programme type, this has been one of many outcomes and used to compare interventions (e.g., diet types), with few studies included. Picot et al. (2012) summarised weight gain in different programme types without meta-analysis.^12^ The three studies they included gave conflicting results on whether weight gain was faster in inpatient or outpatient programmes. Das et al. (2020) included a meta-analysis of weight gain by programme type.^13^ However, only one study was included.

While previous Sphere standards for therapeutic nutrition programmes included guidance on weight gain targets, the latest editions do not.^14^^,^^15^ In 2023, WHO released guidelines on the management of children with wasting and oedematous malnutrition.^16^ They did not set out to review evidence on optimal weight gain, however, they remarked that “150–185 kcal/kg/day should be provided as a starting quantity for a target weight gain of 5–10 g/kg/d”. Sachdev and Kurpad (2024), who critiqued WHO's estimation of therapeutic energy requirements, instead suggested estimating energy requirements based on “typical (average) weight gain”.^17^

The aim of this systematic review and meta-analysis is to inform policy and programme discussions on optimal rate of weight gain in children being treated for severe malnutrition. Specific objectives are to: describe the rate of in-programme weight gain in different types of treatment programmes (e.g., inpatient, outpatient, hybrid); describe average length of stay (LOS) in different types of treatment programmes and explore any association with rate of weight gain; explore any association between rate of weight gain and programme outcomes (e.g., mortality, recovery); describe how weight gain has been measured and reported by treatment programmes.

## Methods

### Search strategy and selection criteria

We conducted systematic review and meta-analysis. Studies that reported rate of in-programme weight gain among children aged 6–59 months with severe malnutrition during inpatient and/or outpatient treatment with RUTF, therapeutic milk, or similar, were eligible for inclusion. Severe malnutrition was defined as WHZ <−3, MUAC <115 mm, or oedematous malnutrition, but other definitions that were appropriate to the study context were accepted, e.g., weight-for-height percentage of median <70%, MUAC <110 mm, weight-for-age z-score <−3, protein-energy malnutrition grade III. Programmes that met the population inclusion criteria but included broader populations were accepted (e.g., broader age range and/or level of malnutrition). There were no restrictions on study design or publication date, to allow consideration of many programme types. Preprints were eligible, while unpublished and grey literature (e.g., conference abstracts) were excluded, except for Emergency Nutrition Network Field Exchange articles which had relevant high-quality additions that would otherwise have been missed. Non-English language papers were excluded.

We searched three databases through Ovid: Embase Classic + Embase (1947–2024), Global Health (1910–2024), and Ovid MEDLINE(R) and Epub Ahead of Print, In-Process, In-Data-Review & Other Non-Indexed Citations and Daily (1946–2024). We ran the final search on 23rd February 2024. Through citation searching of eligible papers and keyword searching on literature databases, we identified some additional papers. Search terms covered “malnutrition”, “undernutrition”, “wasting”, “marasmus”, “kwashiorkor”, and “oedematous malnutrition”, along with “treatment programmes”, “therapeutic feeding”, “supplementary feeding”, “community-management of acute malnutrition”, “community-based therapeutic care”, and “nutrition rehabilitation unit”. The full search strategy is provided in the Supplementary Materials. All references identified were imported to Endnote X9 for deduplication and two-stage screening. Two investigators independently screened the titles and abstracts. GOD, TNG, KA, MA, MK, and A. Ki were involved in screening. Papers were excluded if they reported data already captured in another included paper. Papers that otherwise met the inclusion criteria for the review but did not report weight gain as grams per kilogram per time were ineligible for meta-analysis.

### Procedures

We extracted data using a standardised form. Summary estimates of weight change in g/kg/time were extracted. Where applicable, different arms from the same study were considered to be separate programmes and reported on separate rows in the data extraction table. Of 126 papers in this review, 104 were eligible for analysis, contributing 168 programmes. We categorised programmes into ten subgroups: inpatient, hybrid, outpatient, daycare, simplified, combined, simplified & combined, supplementary feeding programmes (SFPs), outpatient & SFPs, and inpatient & outpatient programmes (definitions in Supplementary Table S2). Hybrid programmes comprised of children treated firstly as inpatients and then as outpatients, while “inpatient & outpatient programmes” reported across inpatient and outpatient programmes without children necessarily progressing from inpatient to outpatient care.

The primary outcome was average in-programme weight gain, recorded as mean/median weight gain in g/kg/day or as otherwise expressed. In our meta-analysis, we reported mean rate of weight gain in g/kg/day with its 95% confidence interval (CI), excluding programmes that only reported a median. The prespecified additional outcomes were average LOS in programme; average weight gain and LOS in subgroups (e.g., regional, oedematous vs non-oedematous, and all discharged vs recovered only); post-discharge weight gain; programme outcomes (i.e., percentage in-programme mortality, recovery, default, and relapse); in-programme developmental outcomes; diarrhoea and pneumonia, and other health outcomes. Study definitions were used. Other information recorded were: (i) study characteristics—first author's surname, publication year, year and country of study, study design, study duration, type of treatment programme, diet type, how weight gain was reported (mean/median, units, period used, sample of children used, i.e., all children or only those who recovered), discharge criteria, in-programme follow-up and completeness of follow-up, post-programme follow-up; (ii) sample characteristics—sample size, age and malnutrition criteria for inclusion in study (and whether these matched our eligibility criteria strictly), prevalence and treatment of oedematous malnutrition; (iii) measure of uncertainty and sample size associated with recorded outcomes (as above). If unclear, we assumed weight gain and LOS were reported as a mean and for all children in programme. If sample size was unclear, we assumed based on other information when reasonably possible. Where both severe and moderate malnutrition was included, we extracted results for the severely malnourished subgroup where possible (otherwise we extracted the result for the entire sample). GOD conducted data extraction and quality appraisal. Data extraction and quality appraisal were verified by TNG, MK, KA, LO, MA, KM, and EW, meaning that all data was verified by two authors.

### Statistics

Since our study extracted descriptive variables rather than associations, we adapted the NICE quality appraisal checklist for quantitative studies reporting correlations and associations, removing irrelevant questions.^18^ This checklist comprised of five sections, including eight questions. Questions related to the population, setting, outcome measurement, analytical methods, and summarising internal and external validity. No studies were excluded based on quality.

Analyses were conducted using STATA (StataCorp. 2023. Release 18. College Station, TX: StataCorp LLC). Meta-analysis investigated the rate of weight gain by programme type (using the estimate for all children preferentially and only those who recovered in the next instance). For inclusion in meta-analysis, estimates had to note uncertainty and sample size. If confidence intervals were not provided, they were imputed from standard deviations (SDs) or standard errors (SEs) where possible. We conducted meta-analysis of subgroups, i.e., all discharged vs recovered only, and regional subgroups. The same analyses were done for LOS. Lastly, subgroup analysis of weight gain by oedematous/non-oedematous malnutrition was conducted. DerSimonian-Laird random-effect models are reported in-text, with fixed-effect (common-effect inverse-variance) models reported in Supplementary Materials. We investigated heterogeneity between studies using Cochran's Q test and measured heterogeneity using the I^2^ and Tau^2^ statistics. We considered heterogeneity to be high when I^2^ ≥ 75%.

We conducted unadjusted and adjusted random-effects meta-regression, using the restricted maximum likelihood method, to investigate potential associations of weight gain with LOS and/or programme outcomes. Adjusted models accounted for programme type and then default. For analyses including programme outcomes, we preferentially calculated the standard error from confidence intervals, or else estimated the SE of the proportion by √((p(1-p)/n)) (where p = proportion and n = sample size). Proportions were calculated from percentages and where the proportion was zero, we assigned a very low value of 0.0001 to facilitate statistical analysis. We conducted sensitivity analyses by: excluding papers that did not strictly meet our population inclusion criteria; including programmes that reported medians (by imputing means from reported medians using the quantile method proposed by Wan et al. (2014) and imputing 95% CIs from inter-quartile ranges using the method advised by Cochrane Handbook for Systematic Reviews of Interventions)^19^^,^^20^; and restricting to one programme per paper. We also did a sensitivity analysis comparing all children vs recovered only, excluding programmes that had not explicitly stated the population used. We investigated publication bias by programme type due to differences between programmes types, using the Egger's test. Methods were pre-specified in the protocol, registered on PROSPERO (ID CRD42023266472). Reporting is in line with PRISMA (Preferred Reporting Items for Systematic Reviews and Meta-Analyses) guidelines.^21^

### Role of the funding source

The funder of the study had no role in study design, data collection, data analysis, data interpretation, writing of the report, or decision to submit the paper for publication.

## Results

**Fig. 1:**
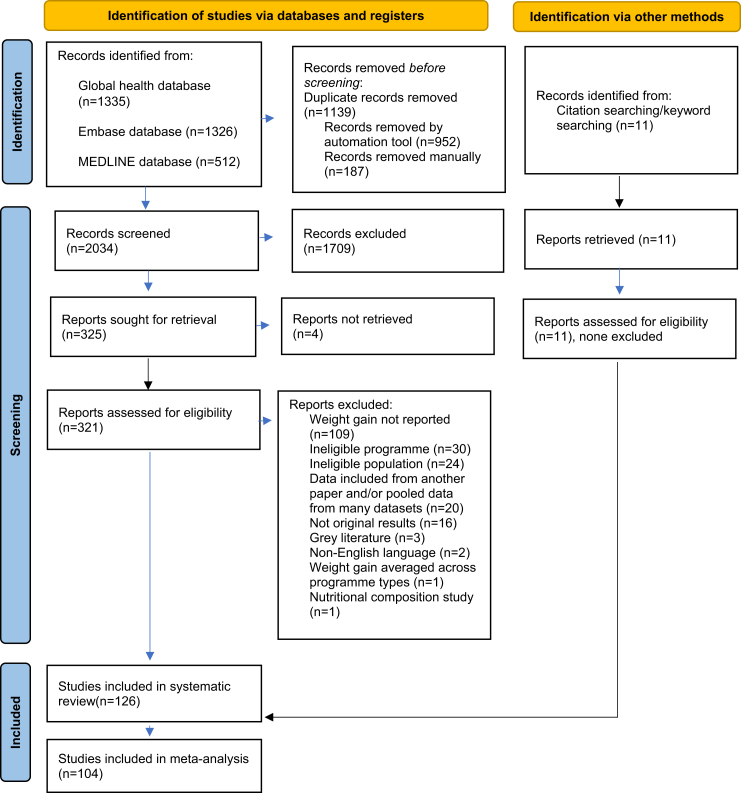
PRISMA diagram.

Our search yielded 3173 papers, of which 1139 were duplicates (Fig. 1). We screened titles and abstracts for the remaining 2034 papers and excluded 1709. This left 325 papers for full text screening; however, four texts were unavailable. We conducted full text screening for the remaining 321 papers and excluded 206 that were ineligible, resulting in 115 papers being identified from our search and screening, with a further 11 identified through other searching, totalling 126 included papers. Twenty-two (16.7%) were ineligible for analysis as they did not report weight gain as g/kg/time (Supplementary Table S3).^22^, ^23^, ^24^, ^25^, ^26^, ^27^, ^28^, ^29^, ^30^, ^31^, ^32^, ^33^, ^34^, ^35^, ^36^, ^37^, ^38^, ^39^, ^40^, ^41^, ^42^, ^43^ Therefore, 104 papers were eligible for analysis, contributing 168 programmes, with some 240,650 participants overall.^44^, ^45^, ^46^, ^47^, ^48^, ^49^, ^50^, ^51^, ^52^, ^53^, ^54^, ^55^, ^56^, ^57^, ^58^, ^59^, ^60^, ^61^, ^62^, ^63^, ^64^, ^65^, ^66^, ^67^, ^68^, ^69^, ^70^, ^71^, ^72^, ^73^, ^74^, ^75^, ^76^, ^77^, ^78^, ^79^, ^80^,^81^, ^82^, ^83^, ^84^, ^85^, ^86^, ^87^, ^88^, ^89^, ^90^, ^91^, ^92^, ^93^, ^94^, ^95^, ^96^, ^97^, ^98^, ^99^, ^100^, ^101^, ^102^, ^103^, ^104^, ^105^, ^106^, ^107^, ^108^, ^109^, ^110^,^111^, ^112^, ^113^, ^114^, ^115^, ^116^, ^117^, ^118^, ^119^, ^120^, ^121^, ^122^, ^123^, ^124^, ^125^, ^126^, ^127^, ^128^, ^129^, ^130^, ^131^, ^132^, ^133^, ^134^, ^135^, ^136^, ^137^, ^138^, ^139^, ^140^, ^141^, ^142^, ^143^, ^144^, ^145^, ^146^, ^147^ Characteristics of these papers are described in Supplementary Table S4. Of the 168 programmes, 32 were inpatient, 18 hybrid, 88 outpatient, 10 daycare, 10 inpatient & outpatient, five simplified & combined, two outpatient & SFP, one simplified, one combined, and one SFP. Most studies (69.1%) were based in Africa while 30.4% were in Asia, and 0.6% were from the Caribbean.

**Fig. 2:**
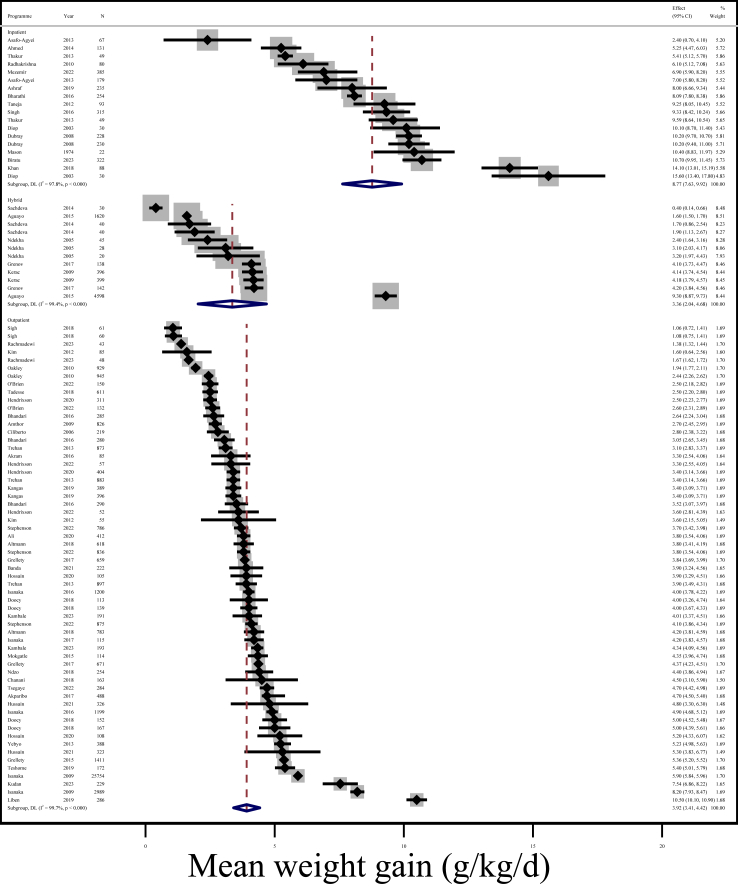
Random-effects meta-analysis of mean weight gain (g/kg/day) by inpatient (n = 18), hybrid (n = 12), and outpatient programmes (n = 60). DL = DerSimonian and Laird; 95% CI = 95% Confidence Interval. NOTE: Weights are from random-effects model.

Overall mean weight gain was 4.8 g/kg/day (95% CI: 4.5, 5.1; I^2^ = 99.8%) across 111 programmes. Fig. 2 shows that mean weight gain was slower in outpatient programmes than inpatient programmes. Mean rate of weight gain was 3.9 g/kg/day (95% CI: 3.4, 4.4; I^2^ = 99.7%) in 60 outpatient programmes, and 8.8 g/kg/day (95% CI: 7.6, 9.9; I^2^ = 97.8%) across 18 inpatient programmes. Over three-quarters (76.5%) of outpatient programmes (65/85) had a mean weight gain <5 g/kg/day. Mean weight gain across 12 hybrid programmes was 3.4 g/kg/day (95% CI: 2.0, 4.7; I^2^ = 99.4%). The forest plot of mean weight gain by programme type is available in Supplementary Figure S1a. Only 10.1% programmes reported weight gain ≥10 g/kg/day, i.e., 15 inpatient, one hybrid, and one outpatient.

Rate of weight gain varied widely, even within programme subgroups. Hybrid programmes varied the most because some reported outpatient and inpatient weight gain separately. For example, the fastest hybrid weight gain was observed in Aguayo et al. (2015): 9.3 g/kg/day (SD = 14.8, n = 4598 children).^45^ This was the inpatient phase, reported separately to the outpatient phase, which had a mean weight gain of 1.6 g/kg/day (SD = 2.0, n = 1620 children). A stark result was the slowest weight gain from Sachdev and Vijayaran (2014), with a very slow mean weight gain of 0.4 g/kg/day (SD = 0.7, n = 30 children).^125^ Children in this programme received only the family diet.

For inpatient, hybrid, and outpatient programmes, weight gain did not differ based on whether all children in-programme were included or only those who recovered (Supplementary Figure S2a and b). In the sensitivity analysis excluding programmes that did not explicitly state which population was used, we also found no difference. Mean weight gain was similar across Africa and Asia, for inpatient, hybrid, and outpatient programmes (Supplementary Figure S3a–c). Although the confidence intervals just overlap, mean weight gain was slower among subgroups with oedematous malnutrition at 4.9 g/kg/day (95% CI: 4.0, 5.7; I^2^ = 96.7%; n = 12 programmes), compared to 6.0 g/kg/day in non-oedematous subgroups (95% CI: 5.5, 6.4; I^2^ = 99.0%; n = 37 programmes) (Supplementary Figure S4a and b). Differences between these subgroups were found in two of three sensitivity analyses (including imputed means and including only those that strictly met inclusion criteria). Only two of eight papers that contributed to the meta-analysis among oedematous subgroups reported accounting for oedema resolution in their calculation of weight gain (e.g., using minimum rather than enrolment weight).

**Fig. 3:**
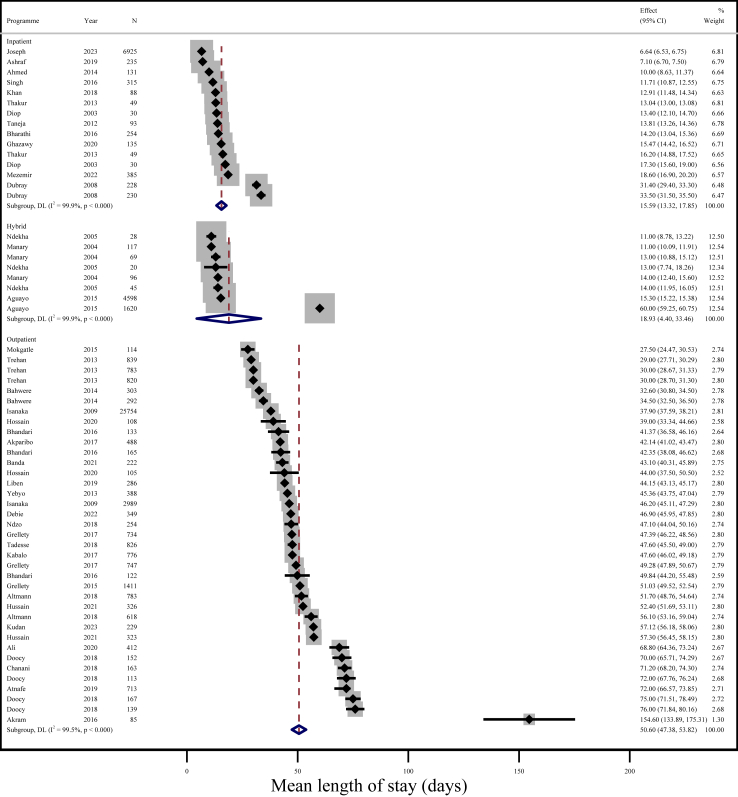
Random-effects meta-analysis of mean length of stay (days) by inpatient (n = 15), hybrid (n = 8), and outpatient programmes (n = 37). DL = DerSimonian and Laird; 95% CI = 95% Confidence Interval. NOTE: Weights are from random-effects model.

Overall mean LOS was 41.1 days (95% CI: 37.9, 44.3; I^2^ = 100%) across 71 programmes. Mean LOS was 50.6 days (95% CI: 47.4, 53.8; I^2^ = 99.5%) across 37 outpatient programmes, compared to 15.6 days (95% CI: 13.3, 17.9; I^2^ = 99.9%) across 15 inpatient programmes (Fig. 3). Mean LOS was 18.9 days (95% CI: 4.4, 33.5; I^2^ = 99.9%) across eight hybrid programmes. Mean LOS in inpatient and outpatient programmes did not differ based on whether all children in-programme were included or only those who recovered (Supplementary Figure S6a and b). Comparing mean LOS in Africa and Asia, there was no difference in inpatient programmes, but in outpatient programmes mean LOS was shorter in Africa (44.1 days; 95% CI: 40.8, 47.5; I^2^ = 99.4%; n = 23 programmes) than in Asia (61.7 days; 95% CI: 56.9, 66.6; I^2^ = 98.3%; n = 14 programmes) (Supplementary Figure S7a and b). There was strong evidence that greater mean weight gain rate was associated with shorter mean LOS (coefficient = −4.0; 95% CI: −6.1, −2.0; p < 0.001; n = 71 programmes). However, there was no evidence of this association after adjusting for programme type (coefficient = −0.3; 95% CI: −2.3, 1.6; p = 0.8; n = 71 programmes).

**Fig. 4:**
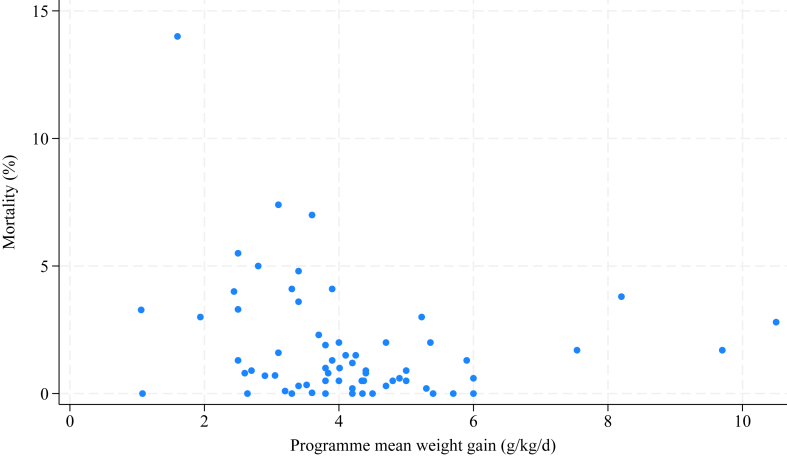
Programme mean weight gain vs percent mortality in outpatient programmes (n = 67 programmes).

Mean mortality was 9.1% across 25 inpatient programmes, while mean mortality was 1.6% across 79 outpatient programmes. There was no evidence for an association between rate of weight gain and percent mortality in unadjusted analysis (coefficient = 0.2; 95% CI: −0.04, 0.5; p = 0.09; n = 118 programmes). After adjusting for programme type, there was some evidence that weight gain was associated with mortality, where a 1 g/kg/day increase in programme mean weight gain was associated with a 0.4 percentage point (pp) reduction in programme mortality (coefficient = −0.4; 95% CI: −0.7, −0.02; p = 0.04; n = 118 programmes). However, there was no evidence of association after further adjusting for default percentage (coefficient = −0.02; 95% CI: −0.4, 0.3; p = 0.9; n = 104 programmes). Findings from the sensitivity analyses were varied, including varying direction of effect. Analysis using only one programme per paper found some evidence that a 1 g/kg/d increase in programme mean weight gain was associated with a 0.3 pp increase in programme mortality (coefficient = 0.3; 95% CI: 0.02, 0.6; p = 0.04; n = 77 programmes) before adjustment for programme type, but no evidence of an association after adjustment (coefficient = −0.2; 95% CI: −0.6, 0.2; p = 0.3; n = 77 programmes). When restricted to programmes strictly meeting population inclusion criteria, there was evidence that a 1 g/kg/d increase in programme mean weight gain was associated with a 0.5 pp increase (coefficient = 0.5; 95% CI: 0.3, 0.7; p < 0.001; n = 75 programmes) and 0.3 pp increase (coefficient = 0.3; 95% CI: 0.02, 0.5; p = 0.04; n = 75 programmes) in programme mortality, before and after adjustment for programme type, respectively. Finally, when imputed means were included, there was no evidence of association in unadjusted analysis (coefficient = 0.2; 95% CI: −0.06, 0.5; p = 0.1; n = 130 programmes), but after adjusting for programme type, there was some evidence for a 0.4 pp reduction in mortality per 1 g/kg/day increase in weight gain after adjusting for programme type (coefficient = −0.4; 95% CI: −0.7, −0.03; p = 0.03; n = 130 programmes). Fig. 4 shows percentage mortality plotted against weight gain in outpatient programmes. The highest mortality was reported by Kim et al. (2012), where the programmes exclusively treated children with HIV.^106^ This was the only outpatient programme (of 79 with relevant data) that exceeded Sphere standards of <10% mortality.^14^ In contrast, 22.2% (18/81) outpatient programmes reported default ≥15% (exceeding Sphere standards).

There was no evidence that weight gain was associated with recovery (n = 117), default (n = 112), or relapse (n = 21), even after adjusting for programme type. However, after further adjustment for default, we found weak evidence for a 1.3 pp increase in recovery per 1 g/kg/day increase in weight gain (coefficient = 1.3; 95% CI: 0.0003, 2.7; p = 0.05; n = 101 programmes).

There was high heterogeneity in all analyses. Quality appraisal showed generally low study-level risk of bias, although this varied between and within studies (Supplementary Table S5). There was no evidence for publication bias accounting for programme types, and no evidence for publication bias in inpatient, hybrid, or outpatient programmes (Egger's test p-values 0.2, 0.3, 0.8, and 0.3, respectively). Sensitivity analyses mostly showed similar results to the main analyses, with any notable differences previously highlighted.

## Discussion

Outpatient programmes reported slower mean weight gain and longer mean LOS than inpatient programmes. They also had low mortality overall. There was inconsistent evidence for an association between weight gain and mortality. We found variable reporting of weight gain and its calculation method. Our results suggest slower weight gain in children with oedematous malnutrition, compared to those without oedema. Only two of eight papers in this analysis explicitly reported accounting for oedema resolution in weight gain calculation.^111^^,^^138^ When oedema resolution is not considered, weight gain appears slower due to the higher baseline weight and longer time period. Therefore, differences may be artificially created.

Our outpatient results are similar to those of Schoonees et al. (2019), who included evidence from some of the same studies as in ours.^148^ They found average weight gain <5 g/kg/day in home-based programmes, in various diet subgroups.^148^ Many factors may explain why this is much lower than the historical 10–15 g/kg/day targets from inpatient programmes. Firstly, food rations may be shared with others.^71^^,^^141^^,^^149^ Second, CMAM programmes emphasise high coverage and proactive case finding.^7^ Many children are thus less severely malnourished at enrolment, and require less catch-up growth. Measurement frequency also impacts weight gain records. Compared to inpatient care, outpatient programmes last longer and have less frequent measurements, e.g., weekly/fortnightly. Therefore, a child in outpatient treatment may have reached discharge target weight several days before this is recorded, resulting in under-estimation of the recorded rate of weight gain. There could be variations due to underlying health status, admission/discharge criteria, and diet also.

As well as being markedly below prior inpatient targets of 10–15 g/kg/day,^6^ most outpatient programmes reported mean weight gain below 2004 Sphere standards recommendation of >8 g/kg/day and WHO's mention of 5–10 g/kg/day.^15^^,^^16^ It is imperative to understand the short-term and long-term risks associated with slower weight gain. Overall mortality was low in most outpatient programmes. We found no association between weight gain and mortality in unadjusted analysis. We found weak evidence for an association between slower programme-level weight gain and increased programme mortality. However, evidence for association and the direction of effect varied in sensitivity analyses. Therefore, this finding may be due to chance, although exploration with individual-level data is warranted. Since most programmes provided similar treatment calories, slow programme-level weight gain may be a red flag for a high burden of more severely wasted or sick children, or may reflect more adverse community conditions or programmatic quality issues. Since this is an observational study where most children were receiving broadly similar treatment, it seems that increasing weight gain through food is unlikely to address the causes of higher mortality risk seen in slow-growing individuals. This is particularly the case as Sachdev and Kurpad (2024) found the WHO guidelines to be an overestimation of therapeutic energy requirements.^17^

A major strength of this review is the comprehensive search, meaning many studies and patients were included. An important evidence gap is thereby addressed. Furthermore, sensitivity analyses are mostly consistent with our main findings. However, we acknowledge limitations. This meta-analysis deals with programme-level data, so results should not be extrapolated to individual patients. Another limitation is that malnutrition outcomes reported in peer-review papers are not necessarily representative of all other programmes. Since research often increases quality of care, weight gain may be lower elsewhere. While we considered diet provided, we do not have information on actual intake. We focused on in-programme mortality, so we cannot comment on post-discharge mortality, which warrants future research. Also defaulting complicates interpretation of in-programme mortality and other outcomes. Furthermore, there was incomplete reporting. Weight gain was not reported as grams/kilogram/time in 17% of papers, and other papers did not provide measures of uncertainty and/or sample size, so could not be included in analysis. Sample size was inferred/assumed where possible. Independent review of data extraction minimised risk of misinterpretations.

More research is required to identify potential differences between subgroups, e.g., children with disabilities and separating by age and sex,^150^ and for other important outcomes, e.g., cognitive development. There was high heterogeneity in our analyses. Clinical heterogeneity may partially explain differences in outcomes between programmes of the same type. There were variations in the populations of the individual programmes, e.g., by age, sex, severity of malnutrition, health status. There was heterogeneity in admission/discharge criteria, weight gain measurement, therapeutic food, feeding regime, length of follow-up, and outcome definitions (e.g., relapse varied by timescale and exact definition). When a population was not exclusively severely malnourished, weight gain was not always reported for the subset who were so affected.^112^^,^^115^ However, these programmes were excluded in sensitivity analysis. Outcomes of hybrid programmes in Aguayo et al. (2015) were reported for the inpatient and outpatient phases separately, explaining variability in the results for hybrid programmes.^45^ Considering this heterogeneity, we conducted random-effects meta-analysis. There was methodological heterogeneity, with variation in study designs. However, we see only minor heterogeneity when comparing the main analysis with sensitivity analyses. Similarly, there is statistical heterogeneity seen by comparing the fixed- and random-effects models, although these differences do not alter our main findings.

The variation in weight gain reporting highlights the need for standardisation. We suggest reporting mean weight gain in g/kg/day, both for all children in-programme and also focussing solely on children who recovered. The calculation would be: (discharge weight (g) minus minimum weight (g)) ÷ (minimum weight (kg) × number of days from minimum weight to discharge). Another useful method could be 1000 × log (discharge weight/minimum weight) ÷ number of days. Using oedema-free, minimum weight is important to accurately capture rate of weight gain in oedematous children. It is important to clearly state which children are included and the discharge criteria used.

Our findings suggest that slower mean weight gain is common and should be expected in outpatient programmes, compared to inpatient programmes. Our study highlights the need for more research, not only to determine the range of optimal weight gain, but also to identify biomarkers for better measurement of health outcomes, rather than relying solely on weight gain as a proxy indicator of health.^151^ Intervention studies with extended follow-up are required to understand if slower, steadier growth might have advantages over rapid weight recovery in the medium- and long-term, as suggested by observational studies.^10^^,^^11^^,^^152^^,^^153^ In the short term, our findings set useful benchmarks for self-evaluation of other programmes.

## Contributors

MK conceptualised the initial study and was responsible for academic supervision and project administration. All authors contributed to the conceptualisation of the expanded current version. DA did an initial version of the review as an MSc thesis. GOD and MK expanded the search strategy and analysis plan, including adding meta-analyses. GOD ran the search and conducted screening and data extraction. TNG, KA, MA, MK, and A. Ki conducted independent screening of sections of the search results. TNG, MK, KA, LO, MA, KM, and EW verified sections of data extraction, meaning that all data were directly accessed and verified by two authors. GOD conducted the analysis and generated the forest plots, with consultation from MK and CO. GOD generated the flow-chart, figures, and tables. GOD drafted a first version of the manuscript. All authors reviewed and commented on the interpretation of the findings. All authors had access to the underlying data in the study and had responsibility for the decision to submit for publication.

## Data sharing statement

All data reported in this study were extracted from published papers. Study protocol is available on PROSPERO. Data and the do-file for analysis is available on the project data repository: https://datacompass.lshtm.ac.uk/id/eprint/4346/.

## Declaration of interests

DT received WHO funding to attend two WHO Guideline Development Group meetings. We have no other competing interests to declare.

## Appendix ASupplementary data

Supplementary Materials

CHANGE Group Collaborators
