# Supplementary material for: Weight gain among children under five with severe malnutrition in therapeutic feeding programmes: a systematic review and meta-analysis
Source: eClinicalMedicine. 2025 Feb 12;81:103083. doi: 10.1016/j.eclinm.2025.103083 (PMC11872456; doi:10.1016/j.eclinm.2025.103083)
Supplement: Supplementary Materials [file mmc1.docx]

Table of Contents

[CHANGE study collaborators group 3](#_Toc185268019)

[Search strategy 3](#_Toc185268020)

[Table S1. Search terms 3](#_Toc185268021)

[Supplementary methods 4](#_Toc185268022)

[Table S2. Classification of programme types 4](#_Toc185268023)

[Supplementary results 5](#_Toc185268024)

[Table S3. Method used to report weight gain for eligible studies that did not report weight gain as grams per kilogram per unit time (n=22). 5](#_Toc185268025)

[Table S4. Study characteristics of 168 programmes from the 104 papers eligible for meta-analysis 7](#_Toc185268026)

[Table S5. Quality appraisal of papers eligible for meta-analysis (n=104) 38](#_Toc185268027)

[Figure S1a. Random-effects meta-analysis of weight gain (g/kg/d) by programme type (n=111 programmes). 40](#_Toc185268028)

[Figure S1b. Fixed-effects meta-analysis of weight gain (g/kg/d) by programme type (n=111 programmes). 41](#_Toc185268029)

[Figure S2a. Random-effects meta-analysis of weight gain (g/kg/d) sub-grouped by programme type, using estimates for all children in-programme (n=68 programmes). 42](#_Toc185268030)

[Figure S2b. Random-effects meta-analysis of weight gain (g/kg/d) sub-grouped by programme type, using estimates for only those children who recovered (n=52 programmes). 43](#_Toc185268031)

[Figure S2c. Fixed-effects meta-analysis of weight gain (g/kg/d) sub-grouped by programme type, using estimates for all children in-programme (n=68 programmes). 44](#_Toc185268032)

[Figure S2d. Fixed-effects meta-analysis of weight gain (g/kg/d) sub-grouped by programme type, using estimates for only those children who recovered (n=52 programmes). 45](#_Toc185268033)

[Figure S3a. Weight gain (g/kg/d) in inpatient programmes by region (random and fixed effects models) (n=18 programmes). 46](#_Toc185268034)

[Figure S3b. Weight gain (g/kg/d) in hybrid programmes by region (random and fixed effects models) (n=12 programmes). 47](#_Toc185268035)

[Figure S3c. Weight gain (g/kg/d) in outpatient programmes by region (random and fixed effects models) (n=60 programmes). 48](#_Toc185268036)

[Figure S4a. Weight gain (g/kg/d) among children with oedematous malnutrition (random and fixed effects models) (n=12 programmes). 49](#_Toc185268037)

[Figure S4b. Weight gain (g/kg/d) among children with non-oedematous malnutrition (random and fixed effects models) (n=37 programmes). 50](#_Toc185268038)

[Figure S5. Fixed-effects meta-analysis of mean length of stay (days) by inpatient (n=15), hybrid (n=8), and outpatient programmes (n=37). 51](#_Toc185268039)

[Figure S6a. Random-effects meta-analysis of mean length of stay (days) by inpatient (n=11) and outpatient programmes (n=17), using estimates for all children in-programme. 52](#_Toc185268040)

[Figure S6b. Random-effects meta-analysis of mean length of stay (days) by inpatient (n=5) and outpatient programmes (n=23), using estimates for only those children who recovered. 53](#_Toc185268041)

[Figure S6c. Fixed-effects meta-analysis of mean length of stay (days) by inpatient (n=11) and outpatient programmes (n=17), using estimates for all children in-programme. 54](#_Toc185268042)

[Figure S6d. Fixed-effects meta-analysis of mean length of stay (days) by inpatient (n=5) and outpatient programmes (n=23), using estimates for only those children who recovered. 55](#_Toc185268043)

[Figure S7a. Mean length of stay (days) in inpatient programmes by region (random and fixed effects models) (n=15 programmes). 56](#_Toc185268044)

[Figure S7b. Mean length of stay (days) in outpatient programmes by region (random and fixed effects models) (n=37 programmes). 57](#_Toc185268045)

[References 58](#_Toc185268046)

# CHANGE study collaborators group

- Suvi T. Kangas
- Gemechu Ameya
- Asha Badaloo

# Search strategy

3 databases searched through Ovid:

- Embase Classic+Embase (1947 to 2024 February 23)
- Global Health (1910 to 2024 Week 8)
- Ovid MEDLINE(R) and Epub Ahead of Print, In-Process, In-Data-Review & Other Non-Indexed Citations and Daily (1946 to February 23, 2024)

This was supplemented by citation searching of eligible papers identified through the search, as well as key word searching on scholarly literature platforms such as the London School of Hygiene and Tropical Medicine library website and Google Scholar.

## Table S1. Search terms

| **Search terms** |
| --- |
| 1. malnutrition.mp. |
| 1. undernutrition.mp. |
| 1. (wasting or wasted).mp. |
| 1. marasmus.mp. |
| 1. kwashiorkor.mp. |
| 1. (“oedematous malnutrition” or “edematous malnutrition”).mp. |
| 1. severe$ malnourish$.mp. |
| 1. acute$ malnourish$.mp. |
| 1. or/1-8 |
| 1. limit 9 to ("infant (1 to 23 months)" or "preschool child (2 to 5 years)") |
| 1. “treatment progra$”.mp. |
| 1. (“therapeutic feeding” or tfp).mp. |
| 1. (“supplementary feeding” or sfp).mp. |
| 1. (“community management of acute malnutrition” or CMAM).mp. |
| 1. (“community-based therapeutic care” or CTC).mp. |
| 1. (“nutrition rehabilitation unit” or nru).mp. |
| 1. “ready to use therapeutic food”.mp. |
| 1. RUTF.mp. |
| 1. (“in patient treatment” or “inpatient treatment”).mp. |
| 1. 19 or “in patient care”.mp. or “inpatient care”.mp. |
| 1. (otp or “outpatient therapeutic progra$).mp. |
| 1. Or/11-21 |
| 1. 10 and 22 |
| 1. Limit 23 to English |

# Supplementary methods

## Table S2. Classification of programme types

|  | Explanation |
| --- | --- |
| Inpatient | Inpatient for duration of treatment |
| Hybrid | Initially treated as inpatients and then transferred to outpatient care to complete treatment |
| Outpatient | Outpatient for duration of treatment |
| Daycare | Treated as outpatients in that they do not stay overnight, but have frequent long visits in clinic/hospital, e.g. 8am-4pm daily |
| Simplified | A simplified approach to traditional outpatient programmes, such as only using mid-upper arm circumference as admission/discharge criteria and/or using a simplified feeding regime |
| Combined | A variation of outpatient treatment programmes that treats children with severe and moderate wasting simultaneously |
| Simplified and combined | A programme with both a simplified and combined approach |
| Supplementary feeding programmes (SFP) | Not aimed at treating children with severe malnutrition but used for this purpose in the case of Moramarco et al (2016) when CMAM and access to hospital was restricted^3^ |
| Outpatient and supplementary feeding programme | A programme that reports both outpatient and SFP together |
| Both inpatient and outpatient | A programme that reports results (e.g. weight gain) across both inpatient and outpatient treatment, but differs from hybrid because the children do not necessarily transfer from inpatient phase to outpatient phase of treatment, e.g. CMAM model |

# Supplementary results

## Table S3. Method used to report weight gain for eligible studies that did not report weight gain as grams per kilogram per unit time (n=22).

| **First author** | **Title** | **Publication date** | **Method used to report weight gain** |
| --- | --- | --- | --- |
| Ahn | Do we need to reconsider the CMAM admission and discharge criteria?; an analysis of CMAM data in South Sudan | 2020 | % weight gain |
| Binns | Safety and practicability of using mid-upper arm circumference as a discharge criterion in community based management of severe acute malnutrition in children aged 6 to 59 months programmes | 2016 | % weight gain |
| Dale | Using mid-upper arm circumference to end treatment of severe acute malnutrition leads to higher weight gains in the most malnourished children | 2013 | % who achieved weight gain of 15% or more |
| Ashraf | Effect of F-75, F-100 and RUTF (ready to use therapeutic food) supplementation in children with severe malnutrition | 2017 | Absolute weight gain |
| Bhargava | Nutritional intervention among rural preschool children--An evaluatory study | 1982 | Absolute weight gain |
| Gueri | A supplementary Feeding Programme for the Management of severe and moderate malnutrition outside hospital | 1985 | Absolute weight gain |
| Hossain | Increased food intake after the addition of amylase rich flour to supplementary food for malnourished children in rural communities of Bangladesh | 2005 | Absolute weight gain |
| Muzaffar | Effectiveness of energy-dense locally made food with micronutrients versus ready to use therapeutic food in complicated severely malnourished children at nutritional stabilization center liaquat university hospital Hyderabad | 2020 | Absolute weight gain |
| Rathore | Efficacy assessment of supplementary food product in management of PEM among children of 3-6 years | 2018 | Absolute weight gain |
| Shragai | Outcomes after Acute Malnutrition Program Adaptations to COVID-19, Uganda, Ethiopia, and Somalia | 2022 | Absolute weight gain |
| Calgaro | Children’s Nutritional Rehabilitation Program in Beira, Mozambique: A Retrospective Study | 2021 | Absolute weight gain |
| Desai | Follow‑up assessment of under‑nourished children under Integrated Child Development Services Scheme in Tapi District, India | 2014 | Absolute weight gain |
| Nohwar | Role of integrated therapy and nutritional counselling in treating malnourished children in M-East ward Mumbai, India: A Longitudinal Study | 2021 | Absolute weight gain |
| Salahuddin | Determine the outcomes of F100 therapeutic feed in children with severe acute malnutrition | 2020 | Absolute weight gain |
| Saleem | Effectiveness of ready-to-use therapeutic food in improving the developmental potential and weight of children aged under five with severe acute malnourishment in Pakistan: A pretest-post test study | 2021 | Absolute weight gain and proportion that gained >15% of baseline weight |
| Saleem | High-dose vitamin D3 in the treatment of severe acute malnutrition: a multicenter double-blind randomized controlled trial | 2018 | Absolute weight gain and proportion that gained >15% of baseline weight |
| Raketa Ella | Evaluation of the effectiveness of cost-free nutrition programme on children in Reo Health District, Burkina Faso | 2020 | Absolute weight gain reported for the programme overall (n=370), while weight gain in g/kg/d was reported for a subgroup of 6 cured inpatients |
| Wittmann | Efficiency of the NNRI food mixture as tested on kwashiorkor patients | 1968 | Daily absolute weight gain reported (graphically) |
| Singh | Locally made ready-to-use therapeutic food for treatment of malnutrition: A randomized controlled trial | 2010 | g/kg/d reported for categories of WAZ and month of intervention (no overall given) |
| Greco | Effect of a low-cost food on the recovery and death rate of malnourished children | 2006 | Grams per day |
| Mogendi | Efficacy of mid-upper arm circumference in identification, follow-up and discharge of malnourished children during nutrition rehabilitation | 2015 | Grams per day |
| Saadia | Clinical Audit of Treatment Outcome (WHO Guidelines)in Severe Acute Malnutrition: Experience at the Children’s Hospital and Institute of Child Health, Multan | 2021 | Incorrectly reported? mg/kg/d |

## Table S4. Study characteristics of 168 programmes from the 104 papers eligible for meta-analysis

| **First author** | **Title** | **Publication year** | **Group** | **Difference between programmes in the same paper** | **Country** | **Study design (as reported, otherwise noted as classified by reviewer)** | **Year(s) of study (i.e. treatment)** | **Age** | **Malnutrition inclusion criteria in paper** | **Type of programme studied** | **Overall sample size** |
| --- | --- | --- | --- | --- | --- | --- | --- | --- | --- | --- | --- |
| Adem | Treatment outcomes and associated factors in hospitalised children with severe acute malnutrition: A prospective cohort study | 2020 | 1 | N/A | Ethiopia | Prospective cohort study | 2018 | 6-59 months | WHZ<-3 or MUAC<115mm or bilateral nutritional pitting oedema, with any complication | Inpatient | 133 |
| Aguayo | How do the new WHO discharge criteria for the treatment of severe acute malnutrition affect the performance of therapeutic feeding programmes? New evidence from India | 2015 | 1 | Inpatient only | India | Cohort study (classified by reviewer) | 2009-2011 | 6-59 months | Bilateral pitting oedema or MUAC<115mm and/or WHZ⩽-3 of median | Hybrid | 6041 |
| Aguayo | How do the new WHO discharge criteria for the treatment of severe acute malnutrition affect the performance of therapeutic feeding programmes? New evidence from India | 2015 | 2 | Inpatient and outpatient | India | Cohort study (classified by reviewer) | 2009-2011 | 6-59 months | Bilateral pitting oedema or MUAC<115mm and/or WHZ⩽-3 of median | Hybrid | 2376 admitted to inpatient and 1914 discharged to outpatient phase |
| Aguayo | Community management of acute malnutrition (CMAM) programme in Pakistan effectively treats children with uncomplicated severe wasting | 2018 | 1 | N/A | Pakistan | Retrospective case series | 2014 | 6-59 months | MUAC<115mm | Inpatient and outpatient | 32,458 |
| Ahmed | Implementing WHO feeding guidelines for inpatient management of malnourished children | 2014 | 1 | N/A | Pakistan | Cross-sectional, observational study | 2009-2010 | 6-59 months | Severe malnutrition including with complications (marasmus defined as weight-for-height <70% of National Center for Health Statistics growth reference; kwashiorkor as bilateral symmetrical pedal oedema, or oedema of hands or face; marasmic-kwashiorkor was presence of both; moderate malnutrition was weight-for-height between 75% and 80%) | Inpatient | 131 analysed (161 in study but 30 excluded because they left or developed rapid weight gain due to massive oedema) |
| Akparibo | Severe acute malnutrition in children aged under 5 years can be successfully managed in a non‐emergency routine community healthcare setting in Ghana | 2017 | 1 | N/A | Ghana | Retrospective cohort study | 2012-2013 | 6-59 months | MUAC≤115mm and/or bilateral pitting oedema, with appetite and no complications | Outpatient | 488 |
| Akram | Home-based rehabilitation of severely malnourished children using indigenous high-density diet | 2016 | 1 | N/A | Pakistan | Retrospective study using programme data | 2009-2010 | 6-23 months | MUAC<115mm and WHZ<-3 | Outpatient | 123 |
| Ali | Community based management of severe acute malnutrition: the MSF experience from an urban slum setting in Bangladesh | 2020 | 1 | N/A | Bangladesh | Descriptive retrospective study using routinely collected programme data | 2011-2012 | 0-59 months | WHZ<-3, MUAC<115mm, bilateral pitting oedema, or WHZ ≥-3 and <-2 with medical complications | Outpatient | 640 in total (599 in outpatient care) |
| Altmann | Effectiveness of a household water, sanitation and hygiene package on an outpatient program for severe acute malnutrition: A pragmatic cluster-randomized controlled trial in Chad | 2018 | 1 | Usual OTP | Chad | Cluster-randomised controlled trial | 2015-2016 | 6-59 months | WHZ<-3 and/or MUAC<115mm, and/or mild/moderate bilateral oedema, without complications | Outpatient | 758 (in 10 clusters) |
| Altmann | Effectiveness of a household water, sanitation and hygiene package on an outpatient program for severe acute malnutrition: A pragmatic cluster-randomized controlled trial in Chad | 2018 | 2 | OTP + WASH (Water, Sanitation, and Hygiene) kit | Chad | Cluster-randomised controlled trial | 2015-2016 | 6-59 months | WHZ<-3 and/or MUAC<115mm, and/or mild/moderate bilateral oedema, without complications | Outpatient | 845 (in 10 clusters) |
| Amthor | The use of home-based therapy with ready-to use therapeutic food to treat malnutrition in a rural area during a food crisis | 2009 | 1 | N/A | Malawi | Prospective cohort study (classified by reviewer) | 2006 | 6-60 months | Oedema and/or weight-for-height <70% of the reference standard, with appetite | Outpatient | 826 |
| Asafo-Agyei | HIV infection in severely malnourished children in Kumasi, Ghana: a cross-sectional prospective study | 2013 | 1 | HIV seronegative children | Ghana | Cross-sectional, prospective study | 2010 | 3 months to 13 years was inclusion criteria | WHZ<-3 or symmetrical nutritional oedema | Inpatient | 179 |
| Asafo-Agyei | HIV infection in severely malnourished children in Kumasi, Ghana: a cross-sectional prospective study | 2013 | 2 | HIV seropositive children (both exposed and infected) | Ghana | Cross-sectional, prospective study | 2010 | 3 months to 13 years was inclusion criteria | WHZ<-3 or symmetrical nutritional oedema | Inpatient | 67 |
| Ashraf | Day clinic vs. hospital care of pneumonia and severe malnutrition in children under five: a randomised trial | 2019 | 1 | Inpatient | Bangladesh | Parallel randomised clinical trial | 2008-2013 | 2-59 months | WHZ<-3, WAZ<-3, HAZ<-3, and/or MUAC<115mm in children aged 6-59 months, with pneumonia | Inpatient | 235 |
| Ashraf | Day clinic vs. hospital care of pneumonia and severe malnutrition in children under five: a randomised trial | 2019 | 2 | Day care | Bangladesh | Parallel randomised clinical trial | 2008-2013 | 2-59 months | WHZ<-3, WAZ<-3, HAZ<-3, and/or MUAC<115mm in children aged 6-59 months, with pneumonia | Day care outpatient | 235 |
| Atnafe | Time of recovery and associated factors of children with severe acute malnutrition treated at outpatient therapeutic feeding program in Dire Dawa, Eastern Ethiopia | 2019 | 1 | N/A | Ethiopia | Retrospective cohort study | 2013-2016 | 6-59 months | WHZ<-3 or MUAC<110mm or nutritional oedema | Outpatient | 713 |
| Bahwere | Uptake of HIV testing and outcomes within a Community-based Therapeutic Care (CTC) programme to treat Severe Acute Malnutrition in Malawi: a descriptive study | 2008 | 1 | Prospective cohort - HIV-positive (received HIV testing with malnutrition treatment programme) | Malawi | Community-based cohort study - prospective cohort | 2002-2005 | Under 5 years | Not clearly specified | Inpatient and outpatient | 22 |
| Bahwere | Uptake of HIV testing and outcomes within a Community-based Therapeutic Care (CTC) programme to treat Severe Acute Malnutrition in Malawi: a descriptive study | 2008 | 2 | Prospective cohort - HIV-negative (received HIV testing with malnutrition treatment programme) | Malawi | Community-based cohort study - prospective cohort | 2002-2005 | Under 5 years | Not clearly specified | Inpatient and outpatient | 692 |
| Bahwere | Uptake of HIV testing and outcomes within a Community-based Therapeutic Care (CTC) programme to treat Severe Acute Malnutrition in Malawi: a descriptive study | 2008 | 3 | Retrospective cohort - HIV-positive (received HIV testing after discharge from programme) | Malawi | Community-based cohort study - retrospective cohort | 2002-2005 | Under 5 years | Not clearly specified | Inpatient and outpatient | 28 |
| Bahwere | Uptake of HIV testing and outcomes within a Community-based Therapeutic Care (CTC) programme to treat Severe Acute Malnutrition in Malawi: a descriptive study | 2008 | 4 | Retrospective cohort - HIV-negative (received HIV testing after discharge from programme) | Malawi | Community-based cohort study - retrospective cohort | 2002-2005 | Under 5 years | Not clearly specified | Inpatient and outpatient | 1107 |
| Bahwere | Effectiveness of milk whey protein-based ready-to-use therapeutic food in treatment of severe acute malnutrition in Malawian under-5 children: a randomised, double-blind, controlled non-inferiority clinical trial | 2014 | 1 | Standard RUTF | Malawi | Randomised, double-blind, controlled, non-inferiority effectiveness clinical trial | 2010-2011 | 6-59 months | MUAC<110mm or pitting oedema grade 1 or 2, without complications | Outpatient | 292 |
| Bahwere | Effectiveness of milk whey protein-based ready-to-use therapeutic food in treatment of severe acute malnutrition in Malawian under-5 children: a randomised, double-blind, controlled non-inferiority clinical trial | 2014 | 2 | Whey Protein Concentrate RUTF | Malawi | Randomised, double-blind, controlled, non-inferiority effectiveness clinical trial | 2010-2011 | 6-59 months | MUAC<110mm or pitting oedema grade 1 or 2, without complications | Outpatient | 308 (303 analysed as some were excluded due to no baseline data or being older than 59 months) |
| Bahwere | Soya, maize, and sorghum–based ready-to-use therapeutic food with amino acid is as efficacious as the standard milk and peanut paste–based formulation for the treatment of severe acute malnutrition in children: a noninferiority individually randomized controlled efficacy clinical trial in Malawi | 2017 | 1 | Standard RUTF - aged 6-23 months | Malawi | Non-blinded, individually randomised, controlled, non-inferiority, efficacy trial | 2015-2016 | 6-23 months | MUAC<115mm and/or grade 1 or 2 bilateral pitting oedema, without complications | Day care outpatient | 280 |
| Bahwere | Soya, maize, and sorghum–based ready-to-use therapeutic food with amino acid is as efficacious as the standard milk and peanut paste–based formulation for the treatment of severe acute malnutrition in children: a noninferiority individually randomized controlled efficacy clinical trial in Malawi | 2017 | 2 | Standard RUTF - aged 24-59 months | Malawi | Non-blinded, individually randomised, controlled, non-inferiority, efficacy trial | 2015-2016 | 24-59 months | MUAC<115mm and/or grade 1 or 2 bilateral pitting oedema, without complications | Day care outpatient | 166 |
| Bahwere | Soya, maize, and sorghum–based ready-to-use therapeutic food with amino acid is as efficacious as the standard milk and peanut paste–based formulation for the treatment of severe acute malnutrition in children: a noninferiority individually randomized controlled efficacy clinical trial in Malawi | 2017 | 3 | Milk-free soya, maize, and sorghum RUTF - aged 6-23 months | Malawi | Non-blinded, individually randomised, controlled, non-inferiority, efficacy trial | 2015-2016 | 6-23 months | MUAC<115mm and/or grade 1 or 2 bilateral pitting oedema, without complications | Day care outpatient | 262 |
| Bahwere | Soya, maize, and sorghum–based ready-to-use therapeutic food with amino acid is as efficacious as the standard milk and peanut paste–based formulation for the treatment of severe acute malnutrition in children: a noninferiority individually randomized controlled efficacy clinical trial in Malawi | 2017 | 4 | Milk-free soya, maize, and sorghum RUTF - aged 24-59 months | Malawi | Non-blinded, individually randomised, controlled, non-inferiority, efficacy trial | 2015-2016 | 24-59 months | MUAC<115mm and/or grade 1 or 2 bilateral pitting oedema, without complications | Day care outpatient | 171 |
| Bahwere | Soya, maize, and sorghum–based ready-to-use therapeutic food with amino acid is as efficacious as the standard milk and peanut paste–based formulation for the treatment of severe acute malnutrition in children: a noninferiority individually randomized controlled efficacy clinical trial in Malawi | 2017 | 5 | Milk, soya, maize, and sorghum RUTF - aged 6-23 months | Malawi | Non-blinded, individually randomised, controlled, non-inferiority, efficacy trial | 2015-2016 | 6-23 months | MUAC<115mm and/or grade 1 or 2 bilateral pitting oedema, without complications | Day care outpatient | 253 |
| Bahwere | Soya, maize, and sorghum–based ready-to-use therapeutic food with amino acid is as efficacious as the standard milk and peanut paste–based formulation for the treatment of severe acute malnutrition in children: a noninferiority individually randomized controlled efficacy clinical trial in Malawi | 2017 | 6 | Milk, soya, maize, and sorghum RUTF - aged 24-59 months | Malawi | Non-blinded, individually randomised, controlled, non-inferiority, efficacy trial | 2015-2016 | 24-59 months | MUAC<115mm and/or grade 1 or 2 bilateral pitting oedema, without complications | Day care outpatient | 167 |
| Bailey | A simplified, combined protocol versus standard treatment for acute malnutrition in children 6–59 months (ComPAS trial): A cluster-randomized controlled non-inferiority trial in Kenya and South Sudan | 2020 | 1 | Combined, simplified protocol | Kenya, South Sudan | Single-blind cluster-randomised controlled non-inferiority trial | 2017-2018 | 6-59 months | MUAC<125mm and/or oedema (grade 1 or 2), with appetite and no complications | Combined, simplified outpatient | 2061 (12 clusters) |
| Bailey | A simplified, combined protocol versus standard treatment for acute malnutrition in children 6–59 months (ComPAS trial): A cluster-randomized controlled non-inferiority trial in Kenya and South Sudan | 2020 | 2 | Standard protocol | Kenya, South Sudan | Single-blind cluster-randomised controlled non-inferiority trial | 2017-2018 | 6-59 months | MUAC<125mm and/or oedema (grade 1 or 2), with appetite and no complications | Outpatient (OTP and SFP) | 2017 (12 clusters) |
| Banda | Report of a pilot program using a milk-free ready-to-use therapeutic food made from soya, maize, and sorghum to treat severe acute malnutrition | 2021 | 1 | N/A | Malawi | Pilot programme | 2018 | 6-59 months | MUAC<115mm or WHZ<-3 or bilateral pitting oedema grade 1 or 2, with good appetite and no complications | Outpatient | 222 |
| Bekalu | Time to recovery and determinants of uncomplicated severe acute malnutrition among 6 to 59 months children from the outpatient therapeutic feeding program in North Shewa Zone of Oromia Region, Ethiopia: A prospective follow-up study | 2022 | 1 | N/A | Ethiopia | Health post-based prospective follow-up study | 2020-2021 | 6-59 months | Uncomplicated SAM indicated by weight-for-height≤70%, WHZ<-3, and/or MUAC<115mm, and/or oedema (grade 1 or 2) | Outpatient | 423 |
| Bhandari | Efficacy of three feeding regimens for home-based management of children with uncomplicated severe acute malnutrition: a randomised trial in India | 2016 | 1 | Centrally produced RUTF | India | Randomised controlled trial (efficacy trial) | 2012-2015 | 6-59 months | WHZ<-3 and/or oedema of the feet, without complications | Outpatient | 298 enrolled |
| Bhandari | Efficacy of three feeding regimens for home-based management of children with uncomplicated severe acute malnutrition: a randomised trial in India | 2016 | 2 | Locally produced RUTF | India | Randomised controlled trial | 2012-2015 | 6-59 months | WHZ<-3 and/or oedema of the feet, without complications | Outpatient | 307 enrolled |
| Bhandari | Efficacy of three feeding regimens for home-based management of children with uncomplicated severe acute malnutrition: a randomised trial in India | 2016 | 3 | Micronutrient-enriched energy-dense home-prepared foods (A-HPF) where the raw ingredients and recipes were provided | India | Randomised controlled trial | 2012-2015 | 6-59 months | WHZ<-3 and/or oedema of the feet, without complications | Outpatient | 301 enrolled |
| Bharathi | An experience at a tertiary level hospital Nutrition Rehabilitation Centre in management of severe acute malnutrition in children aged between 6-59 months adopting World Health Organization recommendations | 2016 | 1 | N/A | India | Retrospective case study | 2014 | 6-59 months | WHZ<-3 and/or MUAC<115mm, and/or bilateral pitting oedema | Inpatient | 254 |
| Biratu | Achievement of adequate weight gain among infants and children with complicated severe acute malnutrition receiving treatment in therapeutic feeding centers of South Sudanese refugee camps in Ethiopia | 2023 | 1 | N/A | Ethiopia (South Sudanese refugee camps) | Historical cohort study (classified by reviewer) | 2019-2021 | 6-59 months | Severe bilateral pitting oedema or severe wasting (MUAC<115 and/or WHZ<-3), with complications | Inpatient | 332 |
| Budul | Treatment outcome of severe acute malnutrition among children (6-59 months) in outpatient therapeutic feeding program in Gursum District, Somali Region, Ethiopia | 2020 | 1 | N/A | Ethiopia | Facility-based retrospective cross-sectional study | 2016-2018 | 6-59 months | MUAC<115mm or nutritional oedema grade 1 or 2, with appetite and no complications | Outpatient | 350 |
| Burza | Community-based management of severe acute malnutrition in India: new evidence from Bihar | 2015 | 1 | Old criteria for admission and discharge (WHZ and MUAC) | India | Observational, retrospective cohort study | 2009-2010 | 6-59 months | WHZ<-3 and/or MUAC<110mm and/or bilateral oedema | Inpatient and outpatient | 3873 |
| Burza | Community-based management of severe acute malnutrition in India: new evidence from Bihar | 2015 | 2 | New criteria for admission and discharge (MUAC only) | India | Observational, retrospective cohort study | 2010-2011 | 6-59 months | MUAC<115mm and/or bilateral oedema | Inpatient and outpatient | 4401 |
| Cazes | Optimising the dosage of ready-to-use therapeutic food in children with uncomplicated severe acute malnutrition in the Democratic Republic of the Congo: a non-inferiority, randomised controlled trial | 2023 | 1 | Standard CMAM protocol | Democratic Republic of Congo | Randomised controlled non-inferiority trial | 2019-2020 | 6-59 months | Uncomplicated SAM, indicated by MUAC<115mm or WHZ<-3 or nutritional oedema (grade 1 or 2), without complications | Outpatient | 240 |
| Cazes | Optimising the dosage of ready-to-use therapeutic food in children with uncomplicated severe acute malnutrition in the Democratic Republic of the Congo: a non-inferiority, randomised controlled trial | 2023 | 2 | OptiMA protocol | Democratic Republic of Congo | Randomised controlled non-inferiority trial | 2019-2020 | 6-59 months | Uncomplicated SAM, indicated by MUAC<115mm or WHZ<-3 or nutritional oedema (grade 1 or 2), without complications | Combined, simplified outpatient | 242 |
| Chanani | Effectiveness of NGO‐government partnership to prevent and treat child wasting in urban India | 2018 | 1 | N/A | India | Cohort study (classified by reviewer) | 2014-2015 | Under 3 years | WHZ<-3; Note moderate wasting and non-wasted children also included in wider programme | Outpatient | 189 severely wasted |
| Chane | Treatment outcome and associated factors among under-five children with severe acute malnutrition admitted to therapeutic feeding unit in Woldia Hospital, North Ethiopia | 2014 | 1 | N/A | Ethiopia | Hospital record-based retrospective cohort study | 2011-2013 | 0-59 months | SAM (anthropometry NR, although introduction states that SAM is predominantly measured by one or more of the following: WHZ<-3, weight-for-height<70%, MUAC<110mm, and presence of bilateral pitting oedema) | Inpatient | 324 |
| Charle-Cuéllar | Effectiveness and coverage of severe acute malnutrition treatment with a simplified protocol in a humanitarian context in Diffa, Niger | 2023 | 1 | Simplified outpatient protocol | Niger | Non-randomised, community-controlled trial | 2020-2021 | 6-59 months | MUAC<115mm or presence of oedema, without complications | Simplified outpatient | 406 |
| Charle-Cuéllar | Effectiveness and coverage of severe acute malnutrition treatment with a simplified protocol in a humanitarian context in Diffa, Niger | 2023 | 2 | Outpatient (CMAM protocol) | Niger | Non-randomised, community-controlled trial | 2020-2021 | 6-59 months | MUAC<115mm, WHZ<-3, or presence of oedema, without complications | Outpatient | 174 |
| Ciliberto | Home-based therapy for oedematous malnutrition with ready-to-use therapeutic food | 2006 | 1 | N/A | Malawi | Prospective case series | 2003-2004 | 12-60 months | Mild oedema and good appetite | Outpatient | 219 |
| Collins | Outpatient care for severely malnourished children in emergency relief programmes: a retrospective cohort study | 2002 | 1 | N/A | Ethiopia | Retrospective cohort study | 2000-2001 | 6-120 months | Weight-for-height<70% of the reference median or bilateral pitting oedema (no exclusion criteria) | Outpatient | 170 |
| Courtney-Haag | Experiences of the Integrated Management of Acute Malnutrition (IMAM) programme in Nepal: from pilot to scale up | 2020 | 1 | N/A | Nepal | Pilot study and evaluation | 2009-2011 | NR | Severely wasted children without complications (criteria for this not specified) | Inpatient & outpatient | 5609 |
| Daures | New approach to simplifying and optimising acute malnutrition treatment in children aged 6–59 months: the OptiMA single-arm proof-of-concept trial in Burkina Faso | 2020 | 1 | N/A | Burkina Faso | Single-arm proof-of-concept trial | 2017-2018 | 6-59 months | MUAC<125mm or bipedal oedema | Combined, simplified outpatient | 4958 (MUAC<125mm or oedema); 824 cases of SAM |
| Debie | Recovery rate of severe acute malnourished children aged 6–59 months enrolled in outpatient therapeutic program at health posts of Central Gondar zone, Ethiopia | 2022 | 1 | N/A | Ethiopia | Institution-based cross-sectional study | 2018-2021 | 6-59 months | MUAC<115mm or nutritional oedema grade 1 or 2, with good appetite and no complications | Outpatient | 349 |
| Derseh | Co-morbidity, treatment outcomes and factors affecting the recovery rate of under -five children with severe acute malnutrition admitted in selected hospitals from Ethiopia: Retrospective follow up study | 2018 | 1 | N/A | Ethiopia | Retrospective follow up study | 2013-2015 | 1-59 months | Weight-for-height<70% or MUAC<110mm or bilateral pitting oedema | Inpatient | 413 |
| Diop | Comparison of the efficacy of a solid ready-to-use food and a liquid, milk-based diet for the rehabilitation of severely malnourished children: a randomized trial | 2003 | 1 | RUTF | Senegal | Open-label, randomised trial | 2001 | 6-36 months | Defined on admission or after oedema resolved as WHZ<-2 | Inpatient | 35 in total (deaths and defaulters excluded from analysis, so the 30 that recovered were in analysis) |
| Diop | Comparison of the efficacy of a solid ready-to-use food and a liquid, milk-based diet for the rehabilitation of severely malnourished children: a randomized trial | 2003 | 2 | F100 | Senegal | Open-label, randomised trial | 2001 | 6-36 months | Defined on admission or after oedema resolved as WHZ<-2 | Inpatient | 35 in total (deaths and defaulters excluded from analysis, so the 30 that recovered were in analysis) |
| Doocy | Point-of-use water treatment improves recovery rates among children with severe acute malnutrition in Pakistan: results from a site-randomized trial | 2018 | 1 | Control | Pakistan | Site-randomised trial | 2016 | 6-59 months | MUAC<115mm, without oedema and other complications | Outpatient | 219 |
| Doocy | Point-of-use water treatment improves recovery rates among children with severe acute malnutrition in Pakistan: results from a site-randomized trial | 2018 | 2 | Chlorine disinfectant (Aquatabs) | Pakistan | Site-randomised trial | 2016 | 6-59 months | MUAC<115mm, without oedema and other complications | Outpatient | 231 |
| Doocy | Point-of-use water treatment improves recovery rates among children with severe acute malnutrition in Pakistan: results from a site-randomized trial | 2018 | 3 | Flocculent/disinfectant | Pakistan | Site-randomised trial | 2016 | 6-59 months | MUAC<115mm, without oedema and other complications | Outpatient | 231 |
| Doocy | Point-of-use water treatment improves recovery rates among children with severe acute malnutrition in Pakistan: results from a site-randomized trial | 2018 | 4 | Ceramic filter | Pakistan | Site-randomised trial | 2016 | 6-59 months | MUAC<115mm, without oedema and other complications | Outpatient | 220 |
| Dubray | Treatment of severe malnutrition with 2-day intramuscular ceftriaxone vs 5-day amoxicillin | 2008 | 1 | Amoxicillin twice daily for 5 days | Sudan | Unblinded randomised superiority-controlled trial | 2002-2003 | 6-59 months | Weight-for-height percentage index <70% of the reference median and/or bilateral oedema and/or MUAC<110 | Inpatient | 230 |
| Dubray | Treatment of severe malnutrition with 2-day intramuscular ceftriaxone vs 5-day amoxicillin | 2008 | 2 | Intramuscular ceftriaxone once daily for 2 days | Sudan | Unblinded randomised superiority-controlled trial | 2002-2003 | 6-59 months | Weight-for-height percentage index <70% of the reference median and/or bilateral oedema and/or MUAC<110 | Inpatient | 228 |
| Gaboulaud | Could nutritional rehabilitation at home complement or replace centre-based therapeutic feeding programmes for severe malnutrition? | 2007 | 1 | Inpatient | Niger | Cohort study | 2002-2003 | 6-59 months | WHZ<-3 or MUAC<110mm or bilateral pitting oedema with complications | Inpatient | 660 |
| Gaboulaud | Could nutritional rehabilitation at home complement or replace centre-based therapeutic feeding programmes for severe malnutrition? | 2007 | 2 | Hybrid | Niger | Cohort study | 2002-2003 | 6-59 months | WHZ<-3 or MUAC<110mm or bilateral pitting oedema with complications | Hybrid | 937 |
| Gaboulaud | Could nutritional rehabilitation at home complement or replace centre-based therapeutic feeding programmes for severe malnutrition? | 2007 | 3 | Outpatient | Niger | Cohort study | 2002-2003 | 12-59 months | WHZ<-3 or MUAC<110mm without complications | Outpatient | 340 |
| Gebremedhin | Predictors of time-to-recovery from severe acute malnutrition treated in an outpatient treatment program in health posts of Arba Minch Zuria Woreda, Gamo Zone, Southern Ethiopia: A retrospective cohort study | 2020 | 1 | N/A | Ethiopia | Retrospective cohort study | 2016-2018 | 6-59 months | MUAC<110mm or bilateral pitting oedema | Outpatient | 402 |
| Ghazawy | Survival status and mortality predictors among severely malnourished under 5 years of age children admitted to Minia University maternity and children hospital | 2020 | 1 | N/A | Egypt | Retrospective cohort study | 2018 | 6-59 months | WHZ<-3 and/or nutritional oedema | Inpatient | 135 |
| Grellety | Comparison of weight-for-height and mid-upper arm circumference (MUAC) in a therapeutic feeding programme in South Sudan: is MUAC alone a sufficient criterion for admission of children at high risk of mortality? | 2015 | 1 | N/A | South Sudan | Retrospective analysis of routine programme data | 2010 | 6-59 months | WHZ<-3 and/or MUAC<115mm, without complications | Outpatient | 2205 eligible records (2601 records total available in the time period but not all met inclusion criteria) |
| Grellety | Effects of unconditional cash transfers on the outcome of treatment for severe acute malnutrition (SAM): a cluster-randomised trial in the Democratic Republic of the Congo | 2017 | 1 | Control, i.e. standard treatment plus infant and young child feeding (IYCF) counselling | Democratic Republic of Congo | Cluster-randomised controlled trial | 2015 | 6-59 months | MUAC<115mm and/or WHZ <-3 and/or bilateral oedema, without complications | Outpatient | 747 (10 clusters) |
| Grellety | Effects of unconditional cash transfers on the outcome of treatment for severe acute malnutrition (SAM): a cluster-randomised trial in the Democratic Republic of the Congo | 2017 | 2 | Intervention, i.e. std treatment plus infant and young child feeding (IYCF) counselling plus monthly cash supplement (US$40) | Democratic Republic of Congo | Cluster-randomised controlled trial | 2015 | 6-59 months | MUAC<115mm and/or WHZ <-3 and/or bilateral oedema, without complications | Outpatient | 734 (10 clusters) |
| Grenov | Effect of probiotics on diarrhoea in children with severe acute malnutrition: A randomized controlled study in Uganda | 2017 | 1 | Placebo | Uganda | Randomised, double-blind, placebo-controlled trial | 2014-2015 | 6-59 months | MUAC<115mm or WHZ/WLZ<-3 or bipedal pitting oedema | Hybrid | 200 |
| Grenov | Effect of probiotics on diarrhoea in children with severe acute malnutrition: A randomized controlled study in Uganda | 2017 | 2 | Probiotic | Uganda | Randomised, double-blind, placebo-controlled trial | 2014-2015 | 6-59 months | MUAC<115mm or WHZ/WLZ<-3 or bipedal pitting oedema | Hybrid | 200 |
| Hendrixson | Treatment of severe acute malnutrition with oat or standard ready-to-use therapeutic food: a triple-blind, randomised controlled clinical trial | 2020 | 1 | Standard RUTF | Sierra Leone | Triple-blind randomised controlled trial (non-inferiority trial) | 2018-2019 | 6-59 months | Uncomplicated SAM, indicated by WLZ<-3 or MUAC<115mm and/or bipedal oedema, with appetite | Outpatient | 685 |
| Hendrixson | Treatment of severe acute malnutrition with oat or standard ready-to-use therapeutic food: a triple-blind, randomised controlled clinical trial | 2020 | 2 | Oat RUTF | Sierra Leone | Triple-blind randomised controlled trial (non-inferiority trial) | 2018-2019 | 6-59 months | Uncomplicated SAM, indicated by WLZ<-3 or MUAC<115mm and/or bipedal oedema, with appetite | Outpatient | 721 |
| Hendrixson | An Alternative Oat–Containing, Ready-To-Use, Therapeutic Food Does Not Alter Intestinal Permeability or the 16S Ribosomal RNA Fecal Microbiome Configuration Among Children With Severe Malnutrition in Sierra Leone: A Randomized Controlled Trial | 2022 | 1 | Standard RUTF | Sierra Leone | Prospective, randomised, double-blinded, controlled clinical trial | 2021 | 6-59 months | MUAC<115mm and/or presence of bilateral pitting oedema | Outpatient | 59 |
| Hendrixson | An Alternative Oat–Containing, Ready-To-Use, Therapeutic Food Does Not Alter Intestinal Permeability or the 16S Ribosomal RNA Fecal Microbiome Configuration Among Children With Severe Malnutrition in Sierra Leone: A Randomized Controlled Trial | 2022 | 2 | Oat RUTF | Sierra Leone | Prospective, randomised, double-blinded, controlled clinical trial | 2021 | 6-59 months | MUAC<115mm and/or presence of bilateral pitting oedema | Outpatient | 60 |
| Hossain | Acceptability and efficacy of ready-to-use therapeutic food using soy protein isolate in under-5 children suffering from severe acute malnutrition in Bangladesh: a double-blind randomized non-inferiority trial | 2020 | 1 | Standard RUTF | Bangladesh | Double-blind randomised non-inferiority trial | NR | 6-59 months | WHZ<-3, without complications | Outpatient | 130 |
| Hossain | Acceptability and efficacy of ready-to-use therapeutic food using soy protein isolate in under-5 children suffering from severe acute malnutrition in Bangladesh: a double-blind randomized non-inferiority trial | 2020 | 2 | Soy-based RUTF | Bangladesh | Double-blind randomised non-inferiority trial | NR | 6-59 months | WHZ<-3, without complications | Outpatient | 130 |
| Hussain | Efectiveness of management of severe acute malnutrition (SAM) through community health workers as compared to a traditional facility‑based model: a cluster randomized controlled trial | 2021 | 1 | Standard CMAM protocol delivered by lady health workers | Pakistan | Cluster-randomised controlled trial | 2015-2016 | 6-59 months | MUAC<115mm without medical complications | Outpatient | 399 (3 clusters) |
| Hussain | Efectiveness of management of severe acute malnutrition (SAM) through community health workers as compared to a traditional facility‑based model: a cluster randomized controlled trial | 2021 | 2 | Lady health workers delivering outpatient care from their houses | Pakistan | Cluster randomised controlled trial | 2015-2016 | 6-59 months | MUAC<115mm with appetite and no complications | Outpatient | 430 (3 clusters) |
| Ige | Comparative weight gain with ready-to-use therapeutic food in stunted HIV-infected and -uninfected children in a Nigerian Hospital | 2014 | 1 | HIV-negative children | Nigeria | Prospective cohort study (classified by reviewer) | 2011-2012 | 6-60 months | Stunting (HAZ<-3) in addition to WHZ<-3 (can have oedema and/or complications) | Inpatient | 164 |
| Ige | Comparative weight gain with ready-to-use therapeutic food in stunted HIV-infected and -uninfected children in a Nigerian Hospital | 2014 | 2 | HIV-positive children | Nigeria | Prospective cohort study (classified by reviewer) | 2011-2012 | 6-60 months | Stunting (HAZ<-3) in addition to WHZ<-3 (can have oedema and/or complications) | Inpatient | 61 |
| Isanaka | Assessing the Impact of the Introduction of the World Health Organization Growth Standards and Weight-for-Height z-Score Criterion on the Response to Treatment of Severe Acute Malnutrition in Children: Secondary Data Analysis | 2009 | 1 | National Centre for Health Statistics (NCHS) reference (<70%) | Niger | Secondary data analysis of programme data | 2006 | 6-59 months | Weight-for-height <80% (weight gain calculated in those <70%) | Outpatient | 2,989 |
| Isanaka | Assessing the Impact of the Introduction of the World Health Organization Growth Standards and Weight-for-Height z-Score Criterion on the Response to Treatment of Severe Acute Malnutrition in Children: Secondary Data Analysis | 2009 | 2 | WHO growth standards | Niger | Secondary data analysis of programme data | 2006 | 6-59 months | Weight-for-height <80% (but note looked at using WHO growth standards) | Outpatient | 25,754 |
| Isanaka | Routine amoxicillin for uncomplicated severe acute malnutrition in children | 2016 | 1 | Placebo | Niger | Double-blind, randomised, placebo-controlled trial | 2012-2013 | 6-59 months | Uncomplicated SAM, indicated by WHZ<-3 and/or MUAC<115, with appetite and no complications (oedema was considered a complication) | Outpatient | 1200 |
| Isanaka | Routine amoxicillin for uncomplicated severe acute malnutrition in children | 2016 | 2 | Amoxicillin | Niger | Double-blind, randomised, placebo-controlled trial | 2012-2013 | 6-59 months | Uncomplicated SAM, indicated by WHZ<-3 and/or MUAC<115, with appetite and no complications (oedema was considered a complication) | Outpatient | 1199 |
| Isanaka | Outpatient treatment of severe acute malnutrition: response to treatment with a reduced schedule of therapeutic food distribution | 2017 | 1 | N/A | Niger | Non-randomised pilot intervention study | 2014 | 6-59 months | Uncomplicated SAM, indicated by WHZ<-3 and/or MUAC<115mm, without complications | Outpatient | 115 |
| Isanaka | MUAC as the sole discharge criterion from community‐based management of severe acute malnutrition in Burkina Faso | 2019 | 1 | 15% weight gain as discharge criteria | Burkina Faso | Retrospective analysis of routine programme data | 2007-2009 | 6-59 months | MUAC≤118mm or bipedal pitting oedema | Inpatient and outpatient | 24,792 |
| Isanaka | MUAC as the sole discharge criterion from community‐based management of severe acute malnutrition in Burkina Faso | 2019 | 2 | MUAC ≥124mm as discharge criteria | Burkina Faso | Retrospective analysis of routine programme data | 2009-2011 | 6-59 months | MUAC≤118mm or bipedal pitting oedema | Inpatient and outpatient | 26,049 |
| Jadhav | A randomised controlled facility based trial to assess the impact of indigenously prepared ready to use therapeutic food (RUTF) for children with severe acute malnutrition in India | 2016 | 1 | Medical nutrition therapy (locally produced RUTF) | India | Prospective randomised controlled facility-based trial (efficacy trial) | 2011-2013 | 6-59 months | WHZ ≤ -3, with good appetite (those with chronic underlying illnesses were excluded) | Hybrid | 174 allocated to this arm (76 analysed at week 8) |
| James | Low-dose RUTF protocol and improved service delivery lead to good programme outcomes in the treatment of uncomplicated SAM: a programme report from Myanmar | 2015 | 1 | N/A | Myanmar | Programme report | 2009-2010 | 6-59 months | Uncomplicated SAM, indicated by WHZ<-3 and/or MUAC<110mm, without oedema and no complications | Outpatient | 3083 |
| Jima | Recovery rate and treatment outcome in children aged 6-59 months with severe acute malnutrition admitted to outpatient therapeutic feeding, in Ethiopia | 2023 | 1 | N/A | Ethiopia | Facility-based retrospective cohort study | 2014-2016 | 6-59 months | Uncomplicated SAM, indicated by MUAC<110mm and/or WHZ<-3 or presence of bilateral pitting oedema and no complications | Outpatient | 561 |
| Joseph | Time to recovery and its predictors among children 6–59 months with acute malnutrition admitted to community inpatient therapeutic feeding centers in Katsina State, Northwest Nigeria: a retrospective review of health records (2010–2016) | 2023 | 1 | N/A | Nigeria | Retrospective cross-sectional quantitative review of health records | 2010-2016 | 6-59 months | Complicated SAM indicated by MUAC<115mm and/or nutritional oedema | Inpatient | 6925 |
| Kabalo | Treatment outcomes of severe acute malnutrition in children treated within Outpatient Therapeutic Program (OTP) at Wolaita Zone, Southern Ethiopia: retrospective cross-sectional study | 2017 | 1 | N/A | Ethiopia | Retrospective facility-based cross-sectional study | 2014 | 6-59 months | MUAC<115mm, oedema grade 1 or 2, with appetite and no complications (although some admitted children had complications, grade 3 oedema, and/or did not pass the appetite test) | Outpatient | 794 in total (776 with SAM) |
| Kabeta | Factors associated with treatment outcomes of under-five children with severe acute malnutrition admitted to therapeutic feeding unit of Yirgalem Hospital | 2017 | 1 | N/A | Ethiopia | Hospital-based retrospective study | 2013-2015 | Under 59 months | NR (SAM and medical complications or appetite loss) | Inpatient | 196 records reviewed and 191 included |
| Kaleem | Nutritional rehabilitation of severely malnourished children by high density diet in comparison to ready to use therapeutic food | 2014 | 1 | RUTF | Pakistan | Multi-centre parallel randomised controlled trial | 2011-2012 | 6-59 months | Uncomplicated SAM, indicated by MUAC<115mm or bilateral pitting oedema (grade 1 or 2), without complications | Outpatient | 90 |
| Kaleem | Nutritional rehabilitation of severely malnourished children by high density diet in comparison to ready to use therapeutic food | 2014 | 2 | High density diet | Pakistan | Multi-centre parallel randomised controlled trial | 2011-2012 | 6-59 months | Uncomplicated SAM, indicated by MUAC<115mm or bilateral pitting oedema (grade 1 or 2), without complications | Outpatient | 90 |
| Kaleem | Nutritional rehabilitation of severely malnourished children by high density diet in comparison to ready to use therapeutic food | 2014 | 3 | High density diet plus micronutrient supplementation | Pakistan | Multi-centre parallel randomised controlled trial | 2011-2012 | 6-59 months | Uncomplicated SAM, indicated by MUAC<115mm or bilateral pitting oedema (grade 1 or 2), without complications | Outpatient | 90 |
| Kambale | Probiotics for children with uncomplicated severe acute malnutrition (PruSAM study): A randomized controlled trial in the Democratic Republic of Congo | 2023 | 1 | Placebo | Democratic Republic of Congo | Double-blind randomised placebo-controlled trial | 2021-2022 | 6-24 months | Uncomplicated SAM, indicated by MUAC<115mm and/or WLZ≤-3 and/or grade 1 or 2 nutritional oedema, with no complications | Outpatient | 200 (9 defaulted, leaving 191 in the analysis) |
| Kambale | Probiotics for children with uncomplicated severe acute malnutrition (PruSAM study): A randomized controlled trial in the Democratic Republic of Congo | 2023 | 2 | Probiotics | Democratic Republic of Congo | Double-blind randomised placebo-controlled trial | 2021-2022 | 6-24 months | Uncomplicated SAM, indicated by MUAC<115mm and/or WLZ≤-3 and/or grade 1 or 2 nutritional oedema, with no complications | Outpatient | 200 (7 defaulted, leaving 193 in the analysis) |
| Kangas | Impact of reduced dose of ready-to-use therapeutic foods in children with uncomplicated severe acute malnutrition: A randomised non-inferiority trial in Burkina Faso | 2019 | 1 | Reduced RUTF dose | Burkina Faso | Randomised non-inferiority trial | 2016-2018 | 6-59 months | Uncomplicated SAM, indicated by WHZ<-3 and/or MUAC<115mm, positive appetite test, no oedema or complications | Outpatient | 402 treated |
| Kangas | Impact of reduced dose of ready-to-use therapeutic foods in children with uncomplicated severe acute malnutrition: A randomised non-inferiority trial in Burkina Faso | 2019 | 2 | Standard RUTF dose | Burkina Faso | Randomised non-inferiority trial | 2016-2018 | 6-59 months | Uncomplicated SAM, indicated by WHZ<-3 and/or MUAC<115mm, positive appetite test, no oedema or complications | Outpatient | 399 treated (one excluded after randomisation because they didn't meet the admission criteria) |
| Kangas | Effectiveness of acute malnutrition treatment at health center and community levels with a simplified, combined protocol in Mali - An observational cohort study | 2022 | 1 | MUAC<110 and/or oedema | Mali | Observational cohort study | 2018-2021 | 6-59 months | MUAC<115mm and/or oedema (programme admitted MUAC<125 also) | Combined, simplified outpatient | 9710 (total 27800 including moderate wasting) |
| Kerac | Probiotics and prebiotics for severe acute malnutrition (PRONUT study): a double-blind efficacy randomised controlled trial in Malawi | 2009 | 1 | Control | Malawi | Double-blind efficacy randomised controlled trial | 2006-2007 | 5-168 months | Weight-for-height <70% of median, nutritional oedema, or both, MUAC<110mm, or both | Hybrid | 396 |
| Kerac | Probiotics and prebiotics for severe acute malnutrition (PRONUT study): a double-blind efficacy randomised controlled trial in Malawi | 2009 | 2 | Synbiotic | Malawi | Double-blind efficacy randomised controlled trial | 2006-2007 | 5-168 months | Weight-for-height <70% of median, nutritional oedema, or both, MUAC<110mm, or both | Hybrid | 399 |
| Khan | Weight gain in malnourished children on WHO recommended therapeutic feeding formula F-100 | 2018 | 1 | N/A | Pakistan | Descriptive cross-sectional study | 2015-2016 (according to abstract) but 2013-2014 according to the methods section | 6-59 months | WHZ<-3 | Inpatient | 88 |
| Khanum | Controlled trial of three approaches to the treatment of severe malnutrition | 1994 | 1 | Inpatient | Bangladesh | Controlled trial | 1990-1991 | 12-60 months | Weight-for-height<60% and/or oedema | Inpatient | 200 |
| Khanum | Controlled trial of three approaches to the treatment of severe malnutrition | 1994 | 2 | Outpatient day clinic until recovery | Bangladesh | Controlled trial | 1990-1992 | 12-60 months | Weight-for-height<60% and/or oedema | Day care outpatient | 200 |
| Khanum | Controlled trial of three approaches to the treatment of severe malnutrition | 1994 | 3 | Outpatient day clinic for one week and then at-home follow-up | Bangladesh | Controlled trial | 1990-1993 | 12-60 months | Weight-for-height<60% and/or oedema | Day care outpatient | 173 |
| Kim | Prompt initiation of ART with therapeutic food Is associated with improved outcomes in HIV-infected Malawian children with malnutrition | 2012 | 1 | No antiretroviral therapy (ART) within 21 days | Malawi | Retrospective observational study | 2007-2008 | 6-60 months | Weight-for-height<80% median or MUAC<120mm or bilateral pitting oedema | Outpatient | 85 |
| Kim | Prompt initiation of ART with therapeutic food Is associated with improved outcomes in HIV-infected Malawian children with malnutrition | 2012 | 2 | Antiretroviral therapy (ART) within 21 days | Malawi | Retrospective observational study | 2007-2008 | 6-60 months | Weight-for-height<80% median or MUAC<120mm or bilateral pitting oedema | Outpatient | 55 |
| Kudan | Treatment outcomes and predictors of recovery from severe acute malnutrition among children | 2023 | 1 | N/A | Nigeria | Longitudinal study | 2019-2020 | 6-59 months | Uncomplicated SAM, indicated by WHZ<-3 or MUAC<115mm or bilateral pitting oedema of any grade, without complications (although none were admitted based on WHZ) | Outpatient | 229 |
| Liben | Factors associated with child survival in children admitted to outpatient therapeutic program at public health institutions in Afar Regional State, Ethiopia: a prospective cohort study | 2019 | 1 | N/A | Ethiopia | Prospective cohort study | 2017 | 6-59 months | Unclear (mentions that SAM is predominantly measured by one or more of the following: WHZ<-3, WFH<70% of the median, MUAC<110mm, and presence of bilateral pitting oedema) | Outpatient | 286 |
| Mamidi | Hospital based nutrition rehabilitation of severely undernourished children using energy dense local foods | 2010 | 1 | N/A | India | Hospital-based retrospective cohort study | 2001-2005 | Under 60 months | WHZ<-3 or nutritional oedema or risk of severe wasting due to faulty feeding practices | Inpatient | 309 |
| Manary | Home based therapy for severe malnutrition with ready-to-use food | 2004 | 1 | RUTF | Malawi | Non-randomised controlled trial (classified by reviewer) | 2001 | >12 months | NR | Outpatient phase of hybrid programme (except ALOS given for inpatient phase) | 69 |
| Manary | Home based therapy for severe malnutrition with ready-to-use food | 2004 | 2 | RUTF supplement | Malawi | Non-randomised controlled trial (classified by reviewer) | 2001 | >12 months | NR | Outpatient phase of hybrid programme (except average length of stay given for inpatient phase) | 96 |
| Manary | Home based therapy for severe malnutrition with ready-to-use food | 2004 | 3 | Maize/soy flour | Malawi | Non-randomised controlled trial (classified by reviewer) | 2001 | >12 months | NR | Outpatient phase of hybrid programme (except average length of stay given for inpatient phase) | 117 |
| Mangal | Community management of acute malnutrition in Rajasthan, India | 2020 | 1 | N/A | India | Impact evaluation of a programme using a quasi-experimental design | 2018 | 6-59 months | Uncomplicated SAM, indicated by MUAC<115mm and/or WHZ<-3 | Outpatient | 10,344 |
| Mason | Treatment of severe malnutrition in relief | 1974 | 1 | N/A | Ethiopia | Cohort study (classified by reviewer) | NR | NR | Clinical signs of PEM (pitting oedema, wasted buttocks), anorexia, complicating illness, misery, incapacitation, <70% of standard weight-for-height | Inpatient | 44 |
| Maust | Severe and moderate acute malnutrition can be successfully managed with an integrated protocol in Sierra Leone | 2015 | 1 | Combined | Sierra Leone | Cluster-randomised controlled trial | 2013 | 6-59 months | MUAC<125mm or bipedal oedema, with appetite and no complications (SAM defined as MUAC<115mm or oedema) | Combined outpatient | 1100 (majority MAM) |
| Maust | Severe and moderate acute malnutrition can be successfully managed with an integrated protocol in Sierra Leone | 2015 | 2 | Standard protocol | Sierra Leone | Cluster-randomised controlled trial | 2013 | 6-59 months | MUAC<125mm or WHZ<-3 or bipedal oedema, with appetite and no complications (SAM defined as MUAC<115mm or WHZ<-3 or oedema) | Outpatient (OTP and SFP) | 857 (majority SAM) |
| McLennan | Predictors of rate of weight gain in malnourished children within a realimentation programme | 2008 | 1 | N/A | Dominican Republic | Secondary analysis of a prospective programme evaluation | 2004-2006 | NR | Weight-for-height ≤-2 SD of the median (and paediatrician's judgement) | Outpatient (daycare - daily weekday morning visits) | 105 |
| Mengesha | Treatment outcome and factors affecting time to recovery in children with severe acute malnutrition treated at outpatient therapeutic care program | 2016 | 1 | N/A | Ethiopia | Retrospective cohort study | 2011-2013 | 6-59 months | MUAC<110mm or bilateral pitting oedema, with appetite and no complications | Outpatient | 348 |
| Mezemir | Treatment outcome and associated factors of acute malnutrition among children in the therapeutic feeding center of public hospitals in Addis Ababa, Ethiopia: An institutional-based cross-sectional study | 2022 | 1 | N/A | Ethiopia | Institutional-based cross-sectional study using secondary data from medical records | 2016-2019 | 6-59 months | SAM was not an essential inclusion criterion; SAM was defined as WHZ<-3 and/or MUAC<115mm and/or bilateral pitting oedema | Inpatient | 385 |
| Mokgatle | Community-based management programme for treatment of acute child malnutrition using the out-patient therapeutic treatment approach in Dhas district of Ethiopia | 2015 | 1 | N/A | Ethiopia | Quantitative descriptive survey | 2010 | 6-59 months | MUAC<110mm or presence of bilateral pitting oedema | Outpatient | 163 |
| Molton | Efficacy of a community-embedded RUTF programme to treat childhood malnutrition in Kapanga, DRC | 2015 | 1 | N/A | Democratic Republic of Congo | Prospective cohort study to evaluate programme efficacy | 2012-2013 | Up to age 13 | Observable signs of SAM, including oedematous malnutrition (preference of local staff) | Outpatient | 145 in total (but 83 completed the 4 weeks of treatment and met criteria for SAM or MAM at admission, 49 of which were under 5) |
| Moramarco | Community-based management of child malnutrition in Zambia: HIV/AIDS infection and other risk factors on child survival | 2016 | 1 | N/A | Zambia | Community-based retrospective observational study | 2012-2014 | 6-59 months | WHZ/WLZ≤-3, MUAC≤115mm, and/or bilateral pitting oedema | Outpatient SFP | 858 in total, of which 241 had SAM (data for SAM only used in our analysis) |
| Ndekha | Home-based therapy with ready-to-use therapeutic food is of benefit to malnourished, HIV-infected Malawian children | 2005 | 1 | RUTF | Malawi | Non-randomised controlled trial (classified by reviewer) | 2001 | 12-60 months | NR | Outpatient phase of hybrid programme (except average length of stay given for inpatient phase additionally) | 20 |
| Ndekha | Home-based therapy with ready-to-use therapeutic food is of benefit to malnourished, HIV-infected Malawian children | 2005 | 2 | RUTF supplement | Malawi | Non-randomised controlled trial (classified by reviewer) | 2001 | 12-60 months | NR | Outpatient phase of hybrid programme (except average length of stay given for inpatient phase additionally) | 28 |
| Ndekha | Home-based therapy with ready-to-use therapeutic food is of benefit to malnourished, HIV-infected Malawian children | 2005 | 3 | Maize/soy flour | Malawi | Non-randomised controlled trial (classified by reviewer) | 2001 | 12-60 months | NR | Outpatient phase of hybrid programme (except average length of stay given for inpatient phase additionally) | 45 |
| Ndzo | Outcomes of children aged 6–59 months with severe acute malnutrition at the GADO Outpatient Therapeutic Center in Cameroon | 2018 | 1 | N/A | Cameroon | Retrospective cohort study | 2015-2016 | 6-59 months | Uncomplicated SAM, indicated by WHZ<-3 or MUAC<115mm or bilateral pitting oedema, and no complications | Outpatient | 254 |
| Oakley | A ready-to-use therapeutic food containing 10% milk is less effective than one with 25% milk in the treatment of severely malnourished children | 2010 | 1 | 25% milk RUTF | Malawi | Double-blind, randomised, controlled, clinical, quasi-effectiveness trial | 2008-2009 | 6-59 months | WHZ<-3 and/or bipedal pitting oedema, with good appetite (children with chronic illnesses, incl HIV, congenital abnormalities, and cerebral palsy were excluded) | Outpatient | 945 |
| Oakley | A ready-to-use therapeutic food containing 10% milk is less effective than one with 25% milk in the treatment of severely malnourished children | 2010 | 2 | 10% milk RUTF | Malawi | Double-blind, randomised, controlled, clinical, quasi-effectiveness trial | 2008-2009 | 6-59 months | WHZ<-3 and/or bipedal pitting oedema, with good appetite (children with chronic illnesses, incl HIV, congenital abnormalities, and cerebral palsy, were excluded) | Outpatient | 929 |
| O’Brien | Comparing Azithromycin to Amoxicillin in the Management of Uncomplicated Severe Acute Malnutrition in Burkina Faso: A Pilot Randomized Trial | 2022 | 1 | Azithromycin | Burkina Faso | Pilot individual-randomised trial | 2020 | 6-59 months | Uncomplicated SAM, indicated by WHZ<-3 or MUAC<115mm and no complications (with oedema being considered a complication) | Outpatient | 161 |
| O’Brien | Comparing Azithromycin to Amoxicillin in the Management of Uncomplicated Severe Acute Malnutrition in Burkina Faso: A Pilot Randomized Trial | 2022 | 2 | Amoxicillin | Burkina Faso | Pilot individual-randomised trial | 2020 | 6-59 months | Uncomplicated SAM, indicated by WHZ<-3 or MUAC<115mm and no complications (with oedema being considered a complication) | Outpatient | 140 |
| Odei Obeng-Amoako | Concurrently wasted and stunted 6-59 months children admitted to the outpatient therapeutic feeding programme in Karamoja, Uganda: Prevalence, characteristics, treatment outcomes and response | 2020 | 1 | Not concurrently wasted and stunted | Uganda | Retrospective cohort study | 2016-2017 | 6-59 months | WHZ<-3 and/or MUAC<115mm, without complications | Outpatient | 404 |
| Odei Obeng-Amoako | Concurrently wasted and stunted 6-59 months children admitted to the outpatient therapeutic feeding programme in Karamoja, Uganda: Prevalence, characteristics, treatment outcomes and response | 2020 | 2 | Concurrently wasted and stunted | Uganda | Retrospective cohort study | 2016-2017 | 6-59 months | WHZ<-3 and/or MUAC<115mm, without complications | Outpatient | 384 |
| Phelan | Treatment outcomes and associated factors for hospitalization of children treated for acute malnutrition under the OptiMA simplified protocol: a prospective observational cohort in rural Niger | 2023 | 1 | N/A | Niger | Prospective observational cohort study | 2019 | 6-59 months | Uncomplicated MAM or SAM, indicated by MUAC<125mm or mild/moderate oedema without medical complications and passed appetite test | Combined, simplified outpatient | 1112 |
| Puoane | Evaluating the clinical management of severely malnourished children - A study of two rural district hospitals | 2001 | 1 | N/A | South Africa | Retrospective record review | 1997-1998 | 6 weeks-4 years | NR | Inpatient | 76 |
| Rachmadewi | Ready-to-Use Therapeutic Foods (RUTFs) Based on Local Recipes Are as Efficacious and Have a Higher Acceptability than a Standard Peanut-Based RUTF: A Randomized Controlled Trial in Indonesia | 2023 | 1 | Mungbean-milk paste | Indonesia | Individually-randomised controlled trial | 2021 | 6-59 months | Uncomplicated SAM, indicated by WHZ<-3 and/or MUAC<115mm and/or bilateral pitting oedema +/++, who passed the appetite test, without complications/underlying health conditions, no severe anaemia or body weight less than 4kg (and not allergic to RUTF and not received treatment for SAM in last 2 months) | Outpatient | 61 |
| Rachmadewi | Ready-to-Use Therapeutic Foods (RUTFs) Based on Local Recipes Are as Efficacious and Have a Higher Acceptability than a Standard Peanut-Based RUTF: A Randomized Controlled Trial in Indonesia | 2023 | 2 | Peanut-milk paste filled wafer roll | Indonesia | Individually-randomised controlled trial | 2021 | 6-59 months | Uncomplicated SAM, indicated by WHZ<-3 and/or MUAC<115mm and/or bilateral pitting oedema +/++, who passed the appetite test, without complications/underlying health conditions, no severe anaemia or body weight less than 4kg (and not allergic to RUTF and not received treatment for SAM in last 2 months) | Outpatient | 61 |
| Rachmadewi | Ready-to-Use Therapeutic Foods (RUTFs) Based on Local Recipes Are as Efficacious and Have a Higher Acceptability than a Standard Peanut-Based RUTF: A Randomized Controlled Trial in Indonesia | 2023 | 3 | Thick mungbean-milk paste | Indonesia | Individually-randomised controlled trial | 2021 | 6-59 months | Uncomplicated SAM, indicated by WHZ<-3 and/or MUAC<115mm and/or bilateral pitting oedema +/++, who passed the appetite test, without complications/underlying health conditions, no severe anaemia or body weight less than 4kg (and not allergic to RUTF and not received treatment for SAM in last 2 months) | Outpatient | 60 |
| Rachmadewi | Ready-to-Use Therapeutic Foods (RUTFs) Based on Local Recipes Are as Efficacious and Have a Higher Acceptability than a Standard Peanut-Based RUTF: A Randomized Controlled Trial in Indonesia | 2023 | 4 | Soy-milk paste | Indonesia | Individually-randomised controlled trial | 2021 | 6-59 months | Uncomplicated SAM, indicated by WHZ<-3 and/or MUAC<115mm and/or bilateral pitting oedema +/++, who passed the appetite test, without complications/underlying health conditions, no severe anaemia or body weight less than 4kg (and not allergic to RUTF and not received treatment for SAM in last 2 months) | Outpatient | 60 |
| Rachmadewi | Ready-to-Use Therapeutic Foods (RUTFs) Based on Local Recipes Are as Efficacious and Have a Higher Acceptability than a Standard Peanut-Based RUTF: A Randomized Controlled Trial in Indonesia | 2023 | 5 | Peanut-milk paste RUTF (control) | Indonesia | Individually-randomised controlled trial | 2021 | 6-59 months | Uncomplicated SAM, indicated by WHZ<-3 and/or MUAC<115mm and/or bilateral pitting oedema +/++, who passed the appetite test, without complications/underlying health conditions, no severe anaemia or body weight less than 4kg (and not allergic to RUTF and not received treatment for SAM in last 2 months) | Outpatient | 60 |
| Radhakrishna | Composition of weight gain during nutrition rehabilitation of severely under nourished children in a hospital based study from India | 2010 | 1 | N/A | India | Hospital-based longitudinal study | 2005-2007 | 6-60 months | WHZ<-2 | Inpatient | 80 |
| Sachdeva | Nutritional rehabilitation using energy dense local food as ready to use therapeutic food in hospitalized malnourished children: Case for primary prevention at grass root levels | 2014 | 1 | RUTF + family diet | India | Hospital-based interventional study | 2011-2012 | 6-60 months | Protein Energy Malnutrition grade 3 or 4, without complications | Hybrid | 40 |
| Sachdeva | Nutritional rehabilitation using energy dense local food as ready to use therapeutic food in hospitalized malnourished children: Case for primary prevention at grass root levels | 2014 | 2 | RUTF + multivitamin + family diet | India | Hospital-based interventional study | 2011-2012 | 6-60 months | Protein Energy Malnutrition grade 3 or 4, without complications | Hybrid | 40 |
| Sachdeva | Nutritional rehabilitation using energy dense local food as ready to use therapeutic food in hospitalized malnourished children: Case for primary prevention at grass root levels | 2014 | 3 | Family diet | India | Hospital-based interventional study | 2011-2012 | 6-60 months | Protein Energy Malnutrition grade 3 or 4, without complications | Hybrid | 30 |
| Sadler | Improving the management of severe acute malnutrition in an area of high HIV prevalence | 2008 | 1 | N/A | Malawi | Prospective cohort study | 2003-2004 | Over 6 months | Weight-for-height≤70% or WHZ≤-3 and/or bilateral pitting oedema | Hybrid | 1077 analysed (but 1237 in total) |
| Shanka | Recovery rate and determinants in treatment of children with severe acute malnutrition using outpatient therapeutic feeding program in Kamba District, South West Ethiopia | 2015 | 1 | N/A | Ethiopia | Institution-based retrospective longitudinal study | 2011-2013 | Assume 12-59 months | Assume weight-for-height ≤70% below the median, bilateral pitting oedema, or MUAC<110mm (as per introduction) | Outpatient | 771 |
| Shewade | Effectiveness of indigenous ready-to-use therapeutic food in community-based management of uncomplicated severe acute malnutrition: a randomized controlled trial from India | 2013 | 1 | RUTF | India | Randomised controlled trial | 2011 | 6-59 months | WHZ < -3 or MUAC < 115mm without complications | Outpatient | 13 |
| Sigh | Effectiveness of a locally produced, fish-based food product on weight gain among Cambodian children in the treatment of acute malnutrition: A randomized controlled trial | 2018 | 1 | BP100 RUTF | Cambodia | Prospective, randomised, single-blinded, controlled, home-based trial | 2015-2017 | 6-59 months | WHZ≤ -2.8, and/or MUAC≤115mm, and/or presence of oedema, without complications | Outpatient | 61 |
| Sigh | Effectiveness of a locally produced, fish-based food product on weight gain among Cambodian children in the treatment of acute malnutrition: A randomized controlled trial | 2018 | 2 | Fish-based NumTrey | Cambodia | Prospective, randomised, single-blinded, controlled, home-based trial | 2015-2017 | 6-59 months | WHZ≤ -2.8, and/or MUAC≤115mm, and/or presence of oedema, without complications | Outpatient | 60 |
| Simachew | Treatment outcomes and predictors of recovery from severe acute malnutrition among children aged 6–59 months attending an outpatient therapeutic program in Wenago District, Southern Ethiopia | 2020 | 1 | N/A | Ethiopia | Facility-based cross-sectional study | 2017-2018 | 6-59 months | MUAC<110mm or weight-for-height<70% or bilateral pitting oedema, with appetite and no complications (children transferred from inpatient care were excluded) | Outpatient | 554 |
| Singh | Experience and outcome of children with severe acute malnutrition using locally prepared therapeutic diet | 2016 | 1 | N/A | India | Cross-sectional longitudinal study | 2013 | Under 59 months | WHZ<-3 and/or MUAC<115mm, and/or bilateral oedema, with complications (children under 6 months with WLZ<-3 and/or nutritional oedema admitted regardless of complications) | Inpatient | 315 |
| Stephenson | Low linoleic acid foods with added DHA given to Malawian children with severe acute malnutrition improve cognition: a randomized, triple-blinded, controlled clinical trial | 2022 | 1 | Standard RUTF | Malawi | Triple-blind randomised controlled clinical trial | 2017-2020 | 6-59 months | Uncomplicated SAM, indicated by WHZ<-3 and/or MUAC<115mm and/or bilateral pitting oedema, with a positive appetite test | Outpatient | 896 analysed |
| Stephenson | Low linoleic acid foods with added DHA given to Malawian children with severe acute malnutrition improve cognition: a randomized, triple-blinded, controlled clinical trial | 2022 | 2 | High-oleic peanut RUTF with added DHA | Malawi | Triple-blind randomised controlled clinical trial | 2017-2020 | 6-59 months | Uncomplicated SAM, indicated by WHZ<-3 and/or MUAC<115mm and/or bilateral pitting oedema, with a positive appetite test | Outpatient | 809 analysed |
| Stephenson | Low linoleic acid foods with added DHA given to Malawian children with severe acute malnutrition improve cognition: a randomized, triple-blinded, controlled clinical trial | 2022 | 3 | High-oleic peanut RUTF without added DHA | Malawi | Triple-blind randomised controlled clinical trial | 2017-2020 | 6-59 months | Uncomplicated SAM, indicated by WHZ<-3 and/or MUAC<115mm and/or bilateral pitting oedema, with a positive appetite test | Outpatient | 860 analysed |
| Tadesse | An integrated community‐based outpatient therapeutic feeding programme for severe acute malnutrition in rural Southern Ethiopia: Recovery, fatality, and nutritional status after discharge | 2018 | 1 | N/A | Ethiopia | Prospective cohort study | 2011 | 6-59 months | Study includes non-wasted children but here we report outcomes of children with SAM only (MUAC<115 or oedema) | Outpatient | 826 with SAM (1048 overall) |
| Taneja | A study to evaluate the effect of nutritional intervention measures on admitted children in selected nutrition rehabilitation centers of Indore and Ujjain Divisions of the State of Madhya Pradesh (India) | 2012 | 1 | N/A | India | Prospective cohort study (classified by reviewer) | 2008-2009 | 0-60 months | Severely malnourished children, as well as those with moderate and mild malnutrition who have associated complications | Inpatient | 100 (93 analysed after 7 dropped out) |
| Teferi | Treatment outcome of children with severe acute malnutrition admitted to therapeutic feeding centers in Southern Region of Ethiopia | 2010 | 1 | N/A | Ethiopia | Retrospective review of programme reports | 2003-2004 | No age restriction (however majority were 6-59 months) | Weight-for-height <70% of the median, or MUAC<110mm (in children aged 6-59 months), or bilateral pitting oedema, or BMI<16 (in adults) | Inpatient | 13843 in total (11550 with malnutrition documented) |
| Tesfay | Length of stay to recover from severe acute malnutrition and associated factors among under-five years children admitted to public hospitals in Aksum, Ethiopia | 2020 | 1 | N/A | Ethiopia | Retrospective cohort study | 2016-2019 | 0-59 months | Weight-for-length/height <70% and/or bilateral pitting oedema or MUAC<110 (for those aged 6-59 months); Weight-for-length<70% and/or bilateral pitting oedema or visible severe wasting (for those under 6 months or <3kg) | Inpatient | 564 data available (from 585 eligible) |
| Teshome | Time-to-recovery from severe acute malnutrition in children 6–59 months of age enrolled in the outpatient treatment program in Shebedino, Southern Ethiopia: a prospective cohort study | 2019 | 1 | N/A | Ethiopia | Prospective cohort study | 2015 | 6-59 months | Uncomplicated SAM indicated by MUAC<110mm and/or bilateral pitting oedema (grade 1 or 2), with appetite and no complications | Outpatient | 216 |
| Thakur | Locally-prepared ready-to-use therapeutic food for children with severe acute malnutrition: A controlled trial | 2013 | 1 | Locally prepared F100 | India | Non-randomised controlled trial | 2009-2010 | 6-60 months | Weight-for-height<70% or WHZ<-3, bipedal pitting oedema, or MUAC<115mm, with complications and poor appetite | Inpatient | 58 |
| Thakur | Locally-prepared ready-to-use therapeutic food for children with severe acute malnutrition: A controlled trial | 2013 | 2 | Locally prepared RUTF | India | Non-randomised controlled trial | 2010 | 6-60 months | Weight-for-height<70% or WHZ<-3, bipedal pitting oedema, or MUAC<115mm, with complications and poor appetite | Inpatient | 55 |
| Trehan | Antibiotics as part of the management of severe acute malnutrition | 2013 | 1 | Placebo | Malawi | Double-blind, randomised placebo-controlled trial | 2009-2011 | 6-59 months | Oedema and/or WHZ<-3, without complications | Outpatient | 920 |
| Trehan | Antibiotics as part of the management of severe acute malnutrition | 2013 | 2 | Amoxicillin (80-90mg/kg/d) | Malawi | Double-blind, randomised placebo-controlled trial | 2009-2011 | 6-59 months | Oedema and/or WHZ<-3, without complications | Outpatient | 924 |
| Trehan | Antibiotics as part of the management of severe acute malnutrition | 2013 | 3 | Cefdinir (14mg/kg/d) | Malawi | Double-blind, randomised placebo-controlled trial | 2009-2011 | 6-59 months | Oedema and/or WHZ<-3, without complications | Outpatient | 923 |
| Tsegaye | Predictors of time to recovery from uncomplicated severe acute malnutrition among 6–59months children treated in outpatient treatment in health posts of Nagele Arsi district: a retrospective cohort study | 2022 | 1 | N/A | Ethiopia | Institutional-based retrospective cohort study | 2018-2020 | 6-59 months | Uncomplicated SAM, indicated by MUAC<115mm or presence of bilateral pitting oedema (grade 1 or 2) | Outpatient | 357 |
| Yebyo | Outpatient therapeutic feeding program outcomes and determinants in treatment of severe acute malnutrition in Tigray, Northern Ethiopia: A retrospective cohort study | 2013 | 1 | N/A | Ethiopia | Retrospective cohort study | 2008-2012 | 6-59 months | Uncomplicated SAM, indicated by MUAC<110mm and/or weight-for-height<70% or presence of bilateral pitting oedema, without complications | Outpatient | 628 |

CMAM=Community Management of Acute Malnutrition; HIV=Human Immunodeficiency Virus; MUAC=Mid-Upper Arm Circumference; NR=Not Reported; OTP=Outpatient Therapeutic Programme; RUTF=Ready-to-Use Therapeutic Food; SAM=Severe Acute Malnutrition; WHO=World Health Organisation; WHZ=Weight-for-Height Z-score

## Table S5. Quality appraisal of papers eligible for meta-analysis (n=104)

|  | 1.1 | 1.2 | 2.3 | 2.5 | 3.1 | 4.3 | 5.1 | 5.2 |
| --- | --- | --- | --- | --- | --- | --- | --- | --- |
| Adem et al 2020 | + | ++ | na | ++ | + | + | + | + |
| Aguayo et al 2015 | + | ++ | na | ++ | + | + | + | + |
| Aguayo et al 2018 | + | ++ | na | ++ | + | ++ | ++ | + |
| Ahmed et al 2014 | + | ++ | na | ++ | + | + | + | + |
| Akparibo et al 2017 | + | ++ | na | ++ | ++ | + | + | + |
| Akram et al 2016 | ++ | ++ | na | ++ | ++ | ++ | + | + |
| Ali et al 2020 | ++ | ++ | na | ++ | + | ++ | + | + |
| Altmann et al 2018 | + | ++ | ++ | ++ | + | ++ | ++ | + |
| Amthor et al 2009 | ++ | ++ | na | ++ | ++ | + | + | + |
| Asafo-Agyei et al 2013 | ++ | ++ | na | ++ | + | + | + | ++ |
| Atnafe et al 2019 | ++ | ++ | na | ++ | + | + | + | + |
| Atnafe et al 2019 | ++ | ++ | na | ++ | + | + | + | + |
| Bahwere et al 2008 | ++ | ++ | na | ++ | + | + | + | + |
| Bahwere et al 2014 | + | ++ | + | ++ | ++ | + | + | + |
| Bahwere et al 2017 | + | ++ | + | ++ | ++ | + | + | + |
| Bailey et al 2020 | ++ | ++ | + | ++ | ++ | ++ | ++ | + |
| Banda et al 2021 | + | - | na | ++ | ++ | ++ | + | + |
| Bekalu et al 2022 | ++ | ++ | na | ++ | ++ | + | + | + |
| Bhandari et al 2016 | ++ | ++ | + | ++ | + | + | + | + |
| Bharathi et al 2016 | + | + | na | ++ | + | ++ | + | + |
| Biratu et al 2023 | ++ | ++ | na | ++ | ++ | + | + | + |
| Budul et al 2020 | ++ | ++ | na | ++ | + | + | + | + |
| Burza et al 2015 | ++ | ++ | na | ++ | ++ | + | + | + |
| Cazes et al 2023 | ++ | ++ | + | ++ | + | + | + | + |
| Chanani et al 2018 | + | ++ | na | ++ | ++ | ++ | ++ | + |
| Chane et al 2014 | ++ | ++ | na | ++ | + | + | + | + |
| Charle-Cuéllar et al 2023 | ++ | ++ | na | ++ | + | ++ | + | + |
| Ciliberto et al 2006 | ++ | ++ | na | ++ | + | ++ | + | + |
| Collins and Sadler 2002 | ++ | ++ | na | ++ | ++ | ++ | ++ | + |
| Haang et al 2020 | ++ | - | na | ++ | + | + | + | + |
| Daures et al 2020 | ++ | ++ | na | ++ | ++ | ++ | + | + |
| Debie et al 2022 | ++ | ++ | na | ++ | + | + | + | + |
| Derseh et al 2018 | ++ | ++ | na | ++ | + | + | + | + |
| Diop et al 2003 | + | ++ | + | ++ | ++ | ++ | ++ | + |
| Doocy et al 2018 | ++ | ++ | + | ++ | + | ++ | + | + |
| Dubray et al 2008 | + | ++ | + | ++ | ++ | + | + | + |
| Gaboulaud et al 2006 | + | ++ | na | ++ | + | + | + | + |
| Gebremedhin et al 2020 | ++ | ++ | na | ++ | + | + | + | + |
| Ghazawy et al 2020 | ++ | ++ | na | ++ | ++ | + | + | + |
| Grellety et al 2015 | ++ | ++ | na | ++ | + | ++ | + | + |
| Grellety et al 2017 | ++ | ++ | + | ++ | ++ | + | + | + |
| Grenov et al 2017 | ++ | ++ | ++ | ++ | ++ | + | + | ++ |
| Hendrixson et al 2020 | ++ | ++ | ++ | ++ | ++ | ++ | ++ | + |
| Hendrixson et al 2022 | + | ++ | + | ++ | + | + | + | + |
| Hossain et al 2020 | ++ | ++ | + | ++ | ++ | + | + | + |
| Hussain et al 2021 | ++ | ++ | + | ++ | ++ | ++ | ++ | + |
| Ige et al 2014 | + | ++ | na | ++ | ++ | + | + | + |
| Isanaka et al 2009 | + | ++ | na | ++ | ++ | + | + | + |
| Isanaka et al 2016 | + | ++ | + | ++ | + | + | + | + |
| Isanaka et al 2017 | ++ | + | na | ++ | + | + | + | + |
| Isanaka et al 2019 | + | + | na | ++ | + | ++ | + | + |
| Jadhav et al 2016 | + | + | + | ++ | + | - | - | - |
| James et al 2015 | ++ | + | na | ++ | ++ | ++ | ++ | + |
| Jima et al 2023 | ++ | ++ | na | ++ | + | ++ | ++ | + |
| Joseph et al 2023 | ++ | ++ | na | ++ | + | + | + | + |
| Kabalo and Seifu 2017 | ++ | ++ | na | ++ | + | ++ | + | + |
| Kabeta et al 2017 | ++ | - | na | ++ | ++ | + | + | + |
| Kaleem et al 2014 | + | ++ | + | ++ | ++ | ++ | + | + |
| Kambale et al 2023 | ++ | ++ | + | ++ | + | + | + | + |
| Kangas et al 2019 | ++ | ++ | + | ++ | ++ | ++ | + | + |
| Kangas et al 2022 | ++ | ++ | na | ++ | + | ++ | + | + |
| Kerac et al 2009 | ++ | ++ | + | ++ | ++ | ++ | + | + |
| Khan et al 2018 | + | ++ | na | ++ | ++ | + | + | + |
| Khanum et al 1994 | ++ | ++ | + | ++ | + | + | + | + |
| Kim et al 2012 | + | ++ | na | ++ | + | + | + | + |
| Kudan et al 2023 | ++ | ++ | na | ++ | ++ | ++ | + | + |
| Liben et al 2019 | ++ | - | na | ++ | ++ | ++ | ++ | + |
| Mamidi et al 2010 | + | + | na | ++ | ++ | + | + | + |
| Manary et al 2004 | + | ++ | + | ++ | + | ++ | ++ | + |
| Mangal et al 2020 | + | ++ | na | ++ | + | + | + | + |
| Mason et al 1974 | ++ | + | na | ++ | ++ | + | + | + |
| Maust et al 2015 | + | ++ | + | ++ | + | ++ | + | + |
| McLennan et al 2008 | ++ | + | na | ++ | ++ | + | + | + |
| Mengesha et al 2016 | ++ | ++ | na | ++ | ++ | + | + | + |
| Mezemir et al 2022 | ++ | ++ | na | ++ | + | + | + | + |
| Mokgatle and Demisse 2015 | + | ++ | na | ++ | + | ++ | + | + |
| Molton et al 2015 | ++ | ++ | na | ++ | + | + | + | + |
| Moramarco et al 2016 | ++ | ++ | na | ++ | ++ | ++ | ++ | + |
| Ndekha et al 2005 | + | ++ | + | ++ | + | ++ | ++ | + |
| Ndzo et al 2018 | ++ | ++ | na | ++ | ++ | ++ | + | - |
| Oakley et al 2010 | ++ | ++ | + | ++ | ++ | ++ | ++ | + |
| O’Brien et al 2022 | ++ | ++ | ++ | ++ | ++ | + | ++ | - |
| Odei Obeng-Amoako et al 2020 | ++ | ++ | na | ++ | + | + | + | + |
| Phelan et al 2023 | ++ | ++ | na | ++ | ++ | ++ | ++ | + |
| Puone et al 2001 | ++ | ++ | na | ++ | + | ++ | + | + |
| Rachmadewi et al 2023 | ++ | ++ | + | ++ | ++ | + | + | ++ |
| Radhakrishna et al 2010 | + | ++ | na | ++ | ++ | + | + | + |
| Sachdeva et al 2014 | + | + | na | ++ | + | + | + | + |
| Sadler et al 2008 | ++ | ++ | na | ++ | ++ | ++ | ++ | + |
| Shanka et al 2015 | ++ | ++ | na | ++ | + | + | + | + |
| Shewade et al 2013 | ++ | + | + | ++ | ++ | + | + | + |
| Sigh et al 2018 | + | ++ | + | ++ | ++ | + | + | + |
| Simachew et al 2020 | ++ | ++ | na | ++ | + | ++ | + | + |
| Singh et al 2016 | + | + | na | ++ | + | ++ | + | + |
| Stephenson et al 2022 | ++ | ++ | + | ++ | + | - | + | + |
| Tadesse et al 2017 | ++ | ++ | na | ++ | ++ | - | + | + |
| Taneja et al 2012 | + | - | na | ++ | ++ | + | + | + |
| Teferi et al 2010 | ++ | ++ | na | ++ | ++ | + | + | + |
| Tesfay et al 2020 | ++ | ++ | na | ++ | + | + | + | + |
| Teshome et al 2019 | ++ | ++ | na | ++ | ++ | ++ | ++ | + |
| Thakur et al 2013 | + | + | na | ++ | ++ | ++ | ++ | + |
| Trehan et al 2013 | ++ | ++ | + | ++ | + | ++ | + | + |
| Tsegaye et al 2022 | ++ | ++ | na | ++ | + | + | + | + |
| Yebyo et al 2013 | ++ | ++ | na | ++ | + | + | + | - |

na=not applicable

## Figure S1a. Random-effects meta-analysis of weight gain (g/kg/d) by programme type (n=111 programmes).


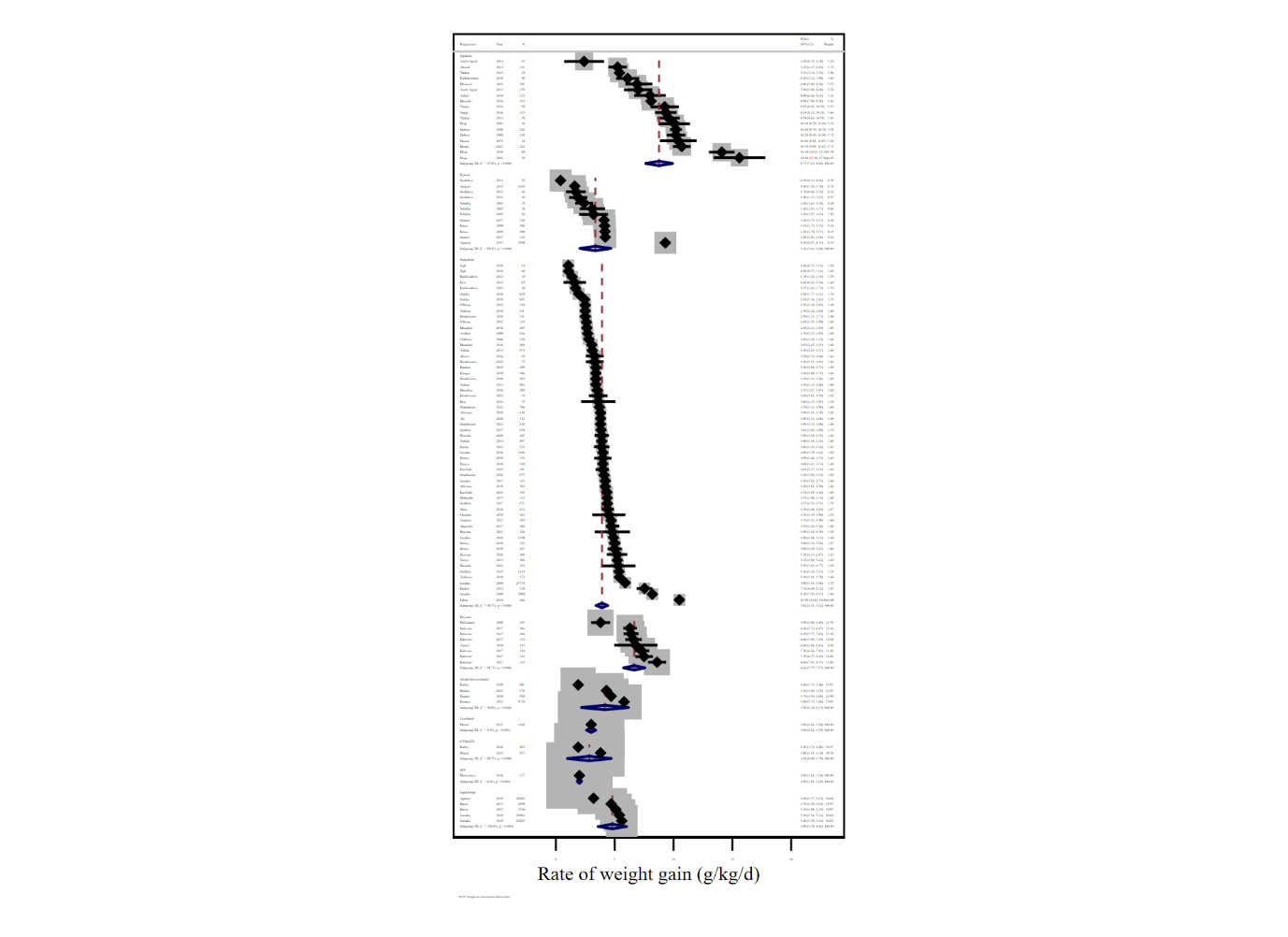


## Figure S1b. Fixed-effects meta-analysis of weight gain (g/kg/d) by programme type (n=111 programmes).


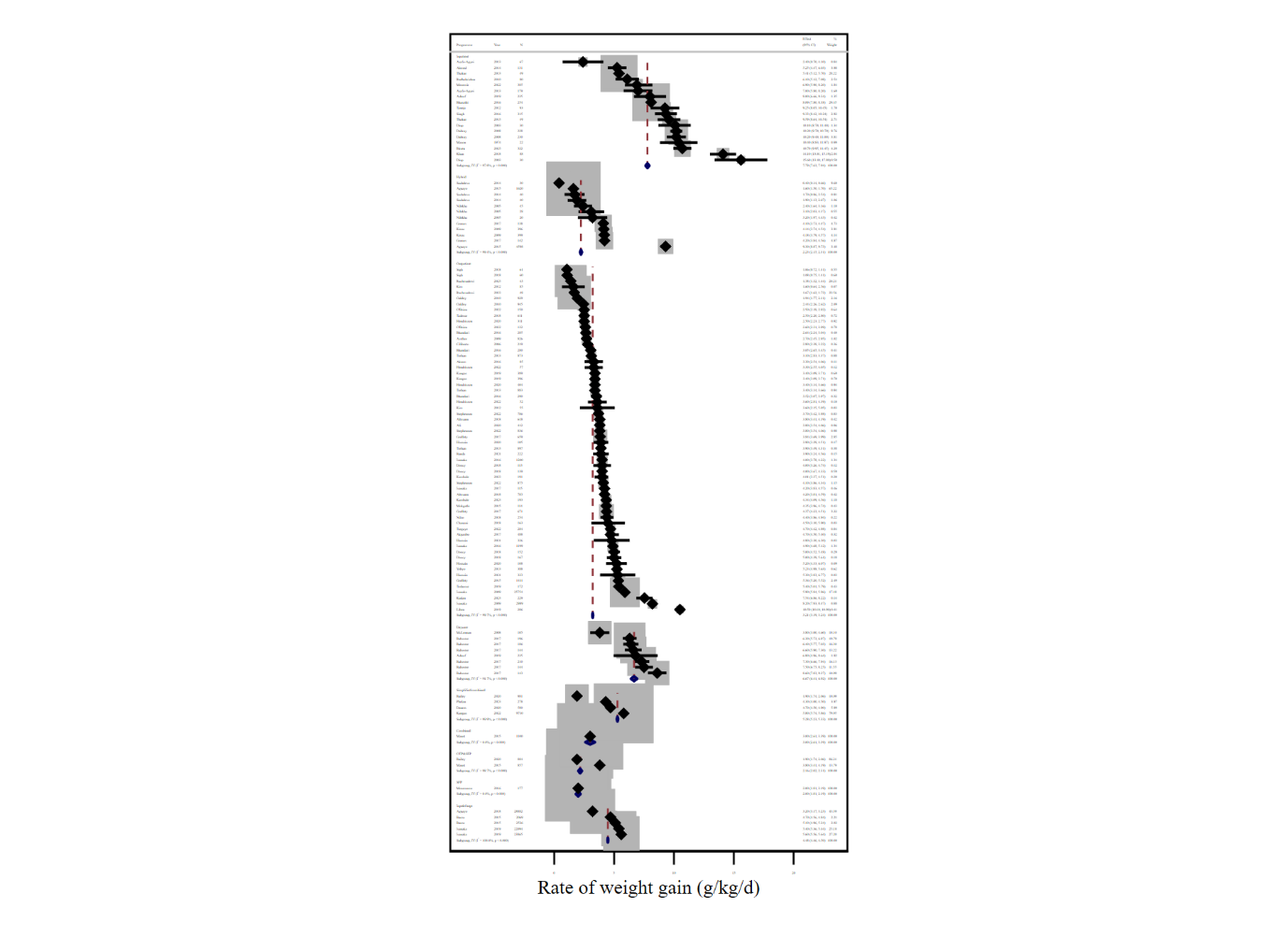


## Figure S2a. Random-effects meta-analysis of weight gain (g/kg/d) sub-grouped by programme type, using estimates for all children in-programme (n=68 programmes).


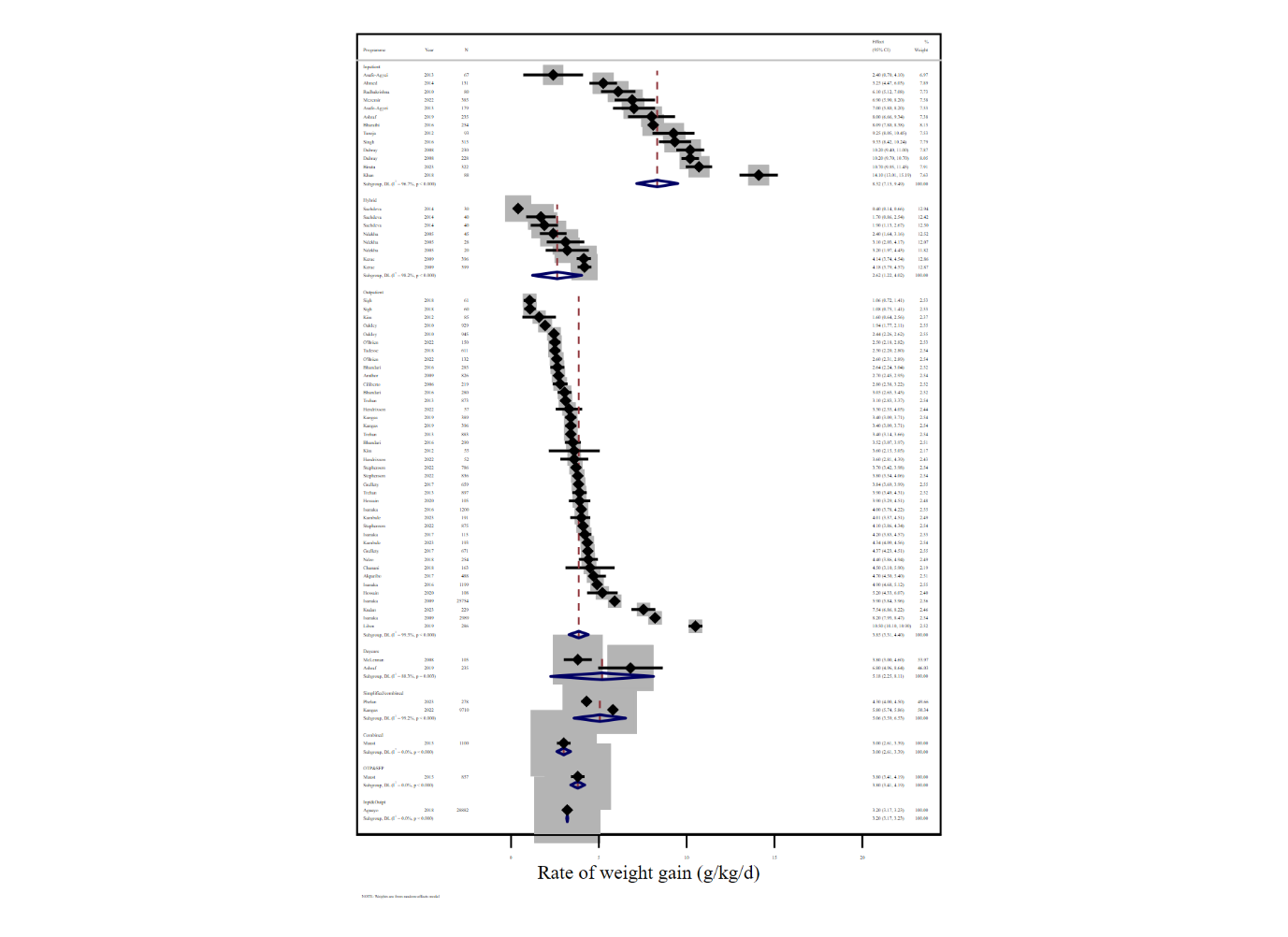


## Figure S2b. Random-effects meta-analysis of weight gain (g/kg/d) sub-grouped by programme type, using estimates for only those children who recovered (n=52 programmes).


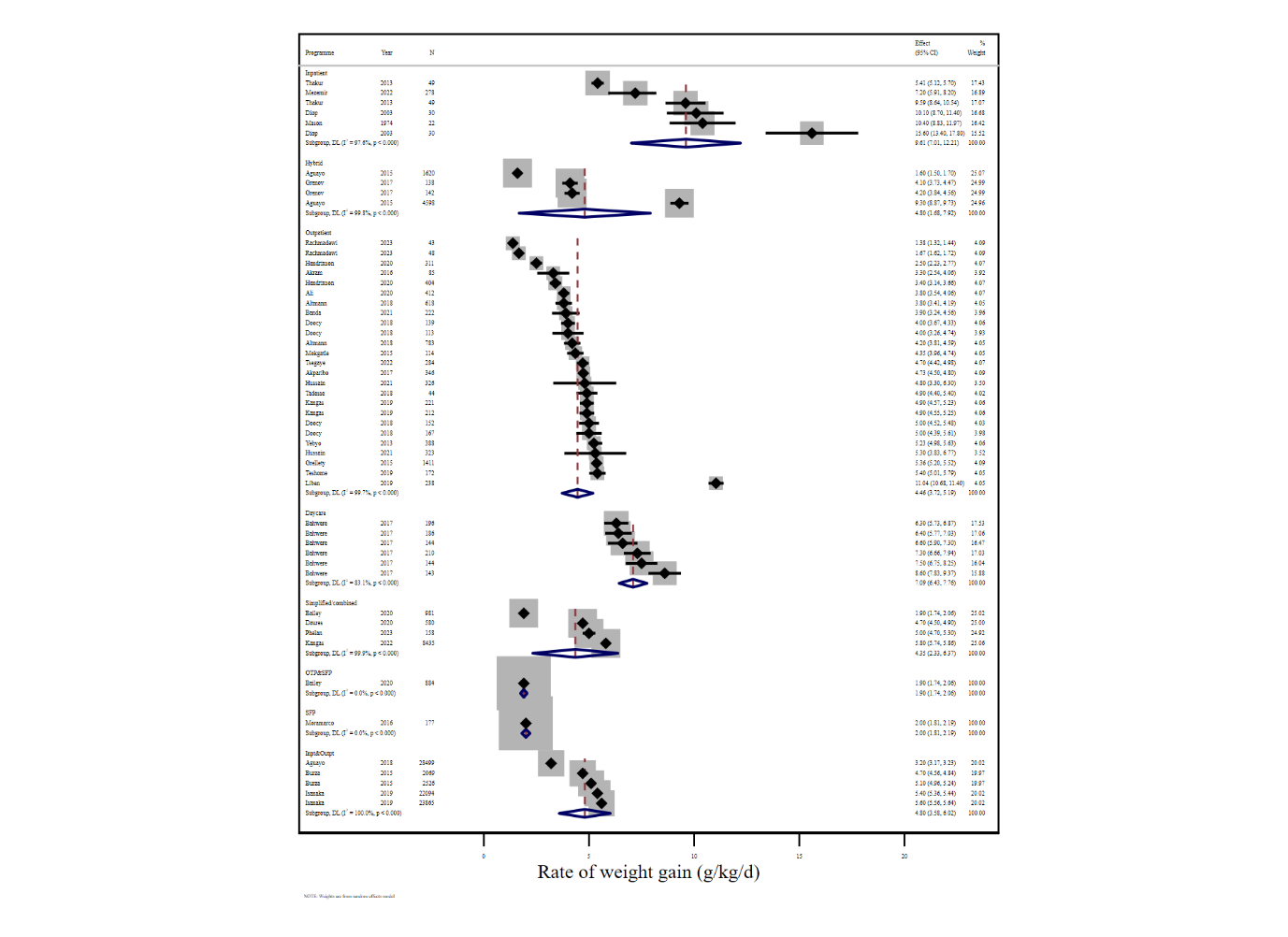


## Figure S2c. Fixed-effects meta-analysis of weight gain (g/kg/d) sub-grouped by programme type, using estimates for all children in-programme (n=68 programmes).


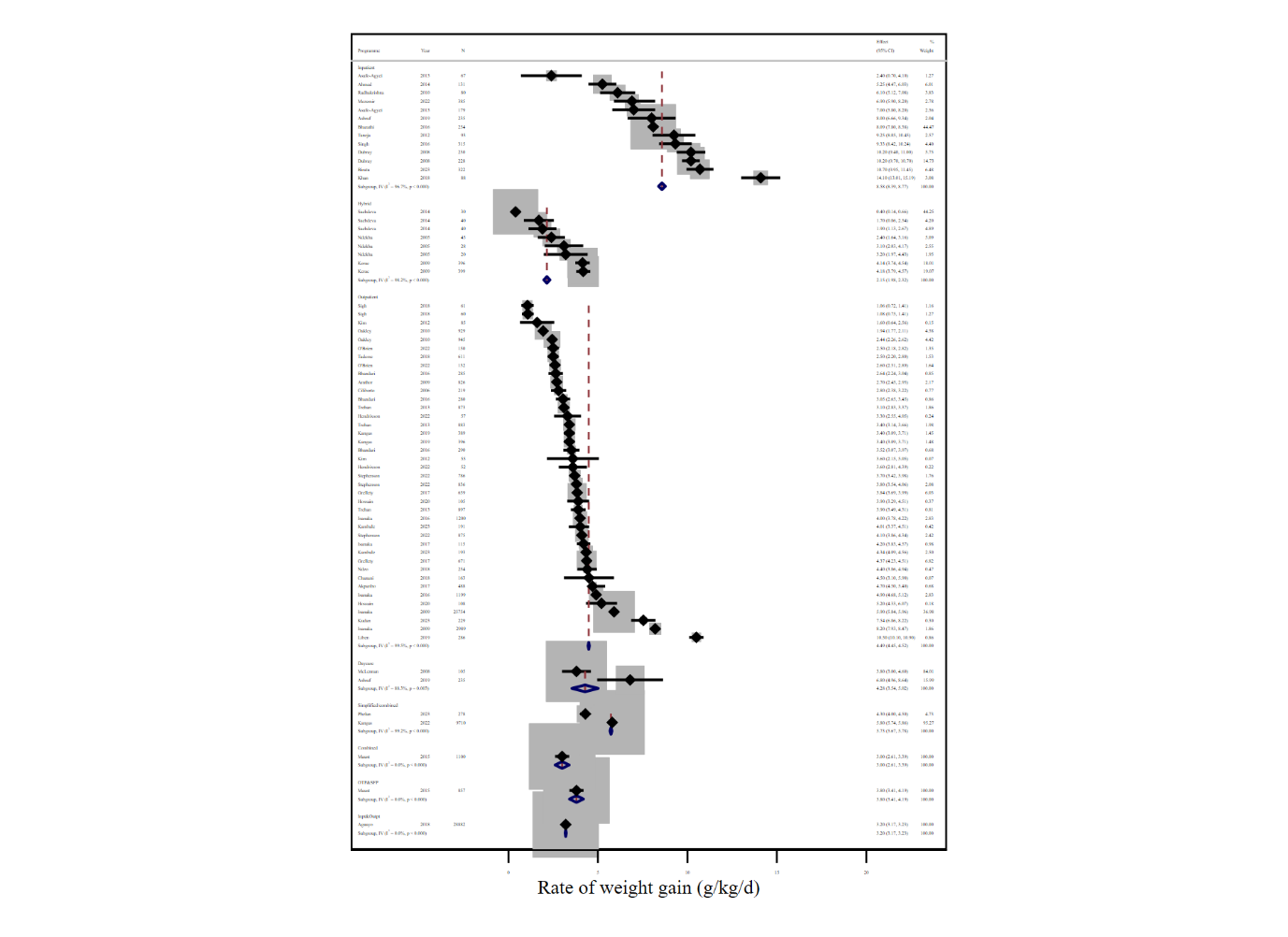


## Figure S2d. Fixed-effects meta-analysis of weight gain (g/kg/d) sub-grouped by programme type, using estimates for only those children who recovered (n=52 programmes).


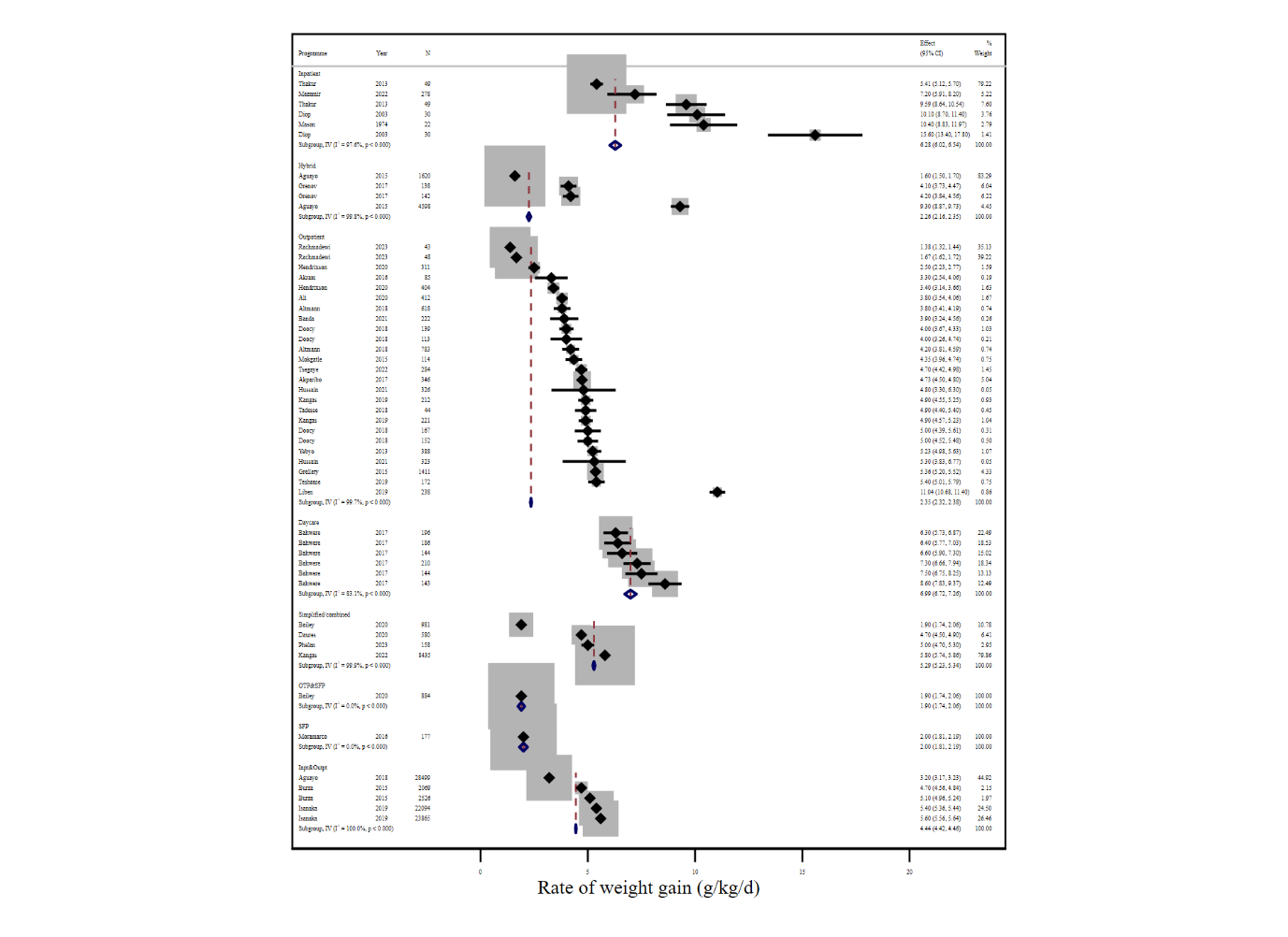


## Figure S3a. Weight gain (g/kg/d) in inpatient programmes by region (random and fixed effects models) (n=18 programmes).


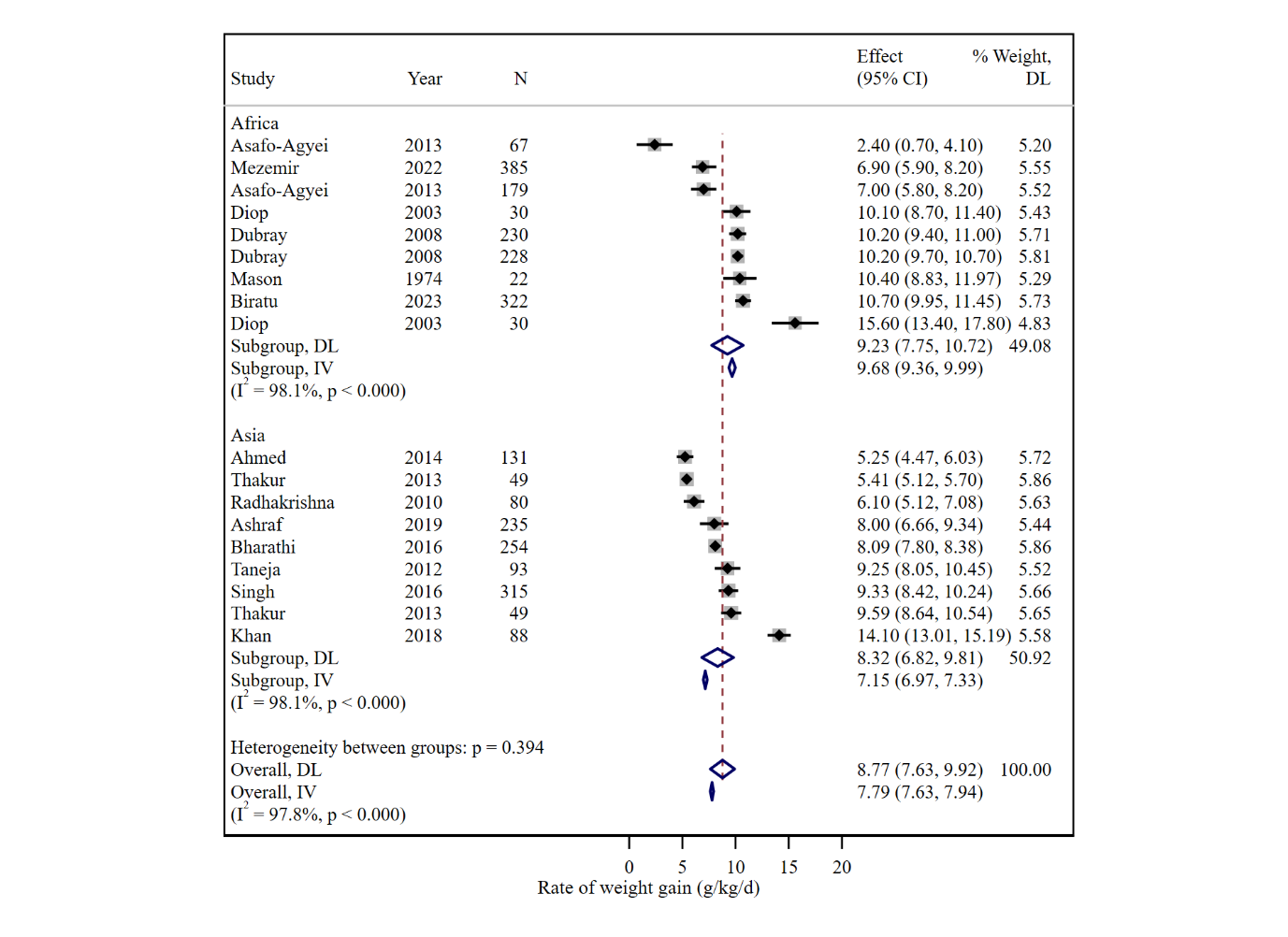


## Figure S3b. Weight gain (g/kg/d) in hybrid programmes by region (random and fixed effects models) (n=12 programmes).


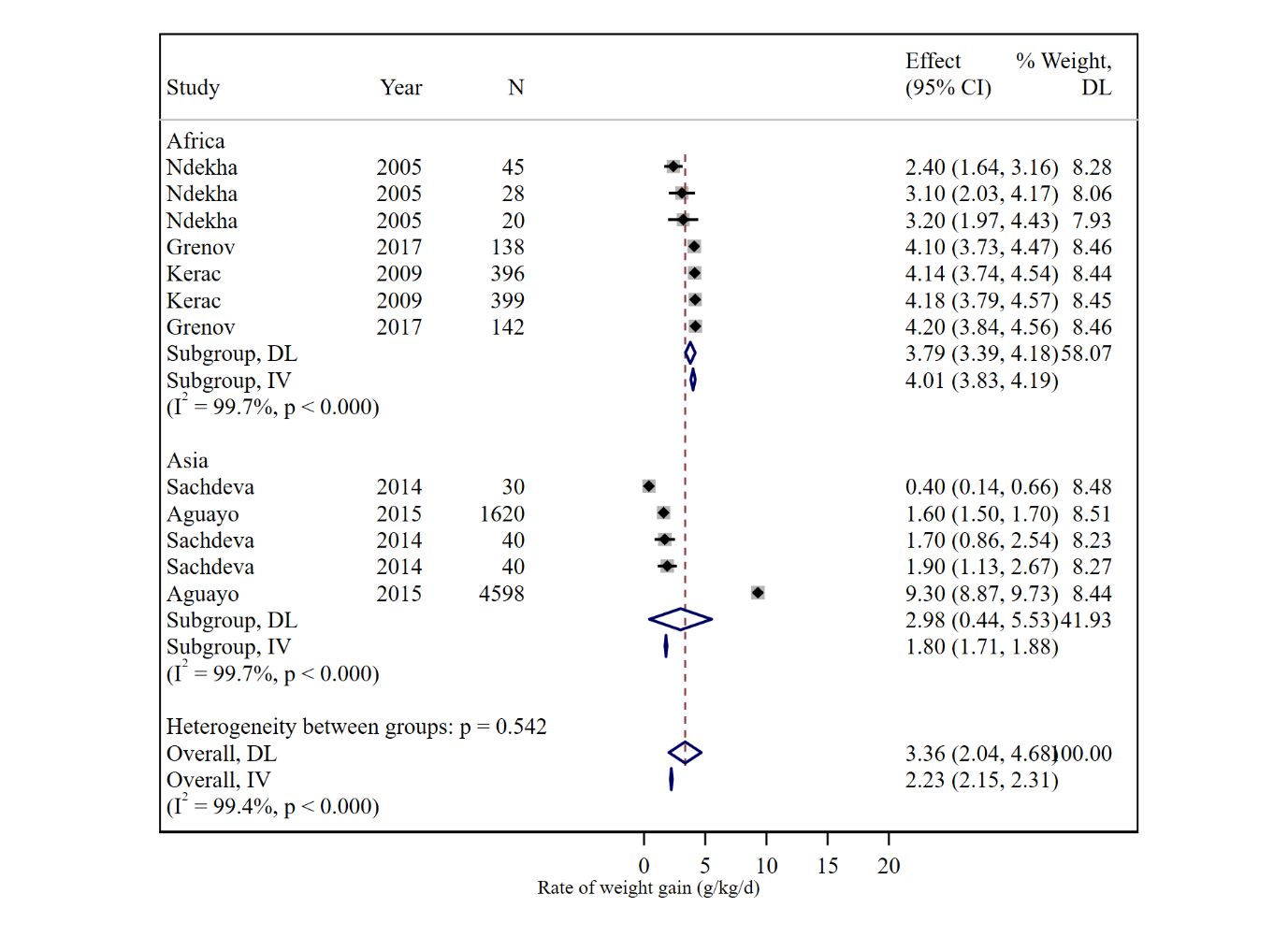


## Figure S3c. Weight gain (g/kg/d) in outpatient programmes by region (random and fixed effects models) (n=60 programmes).


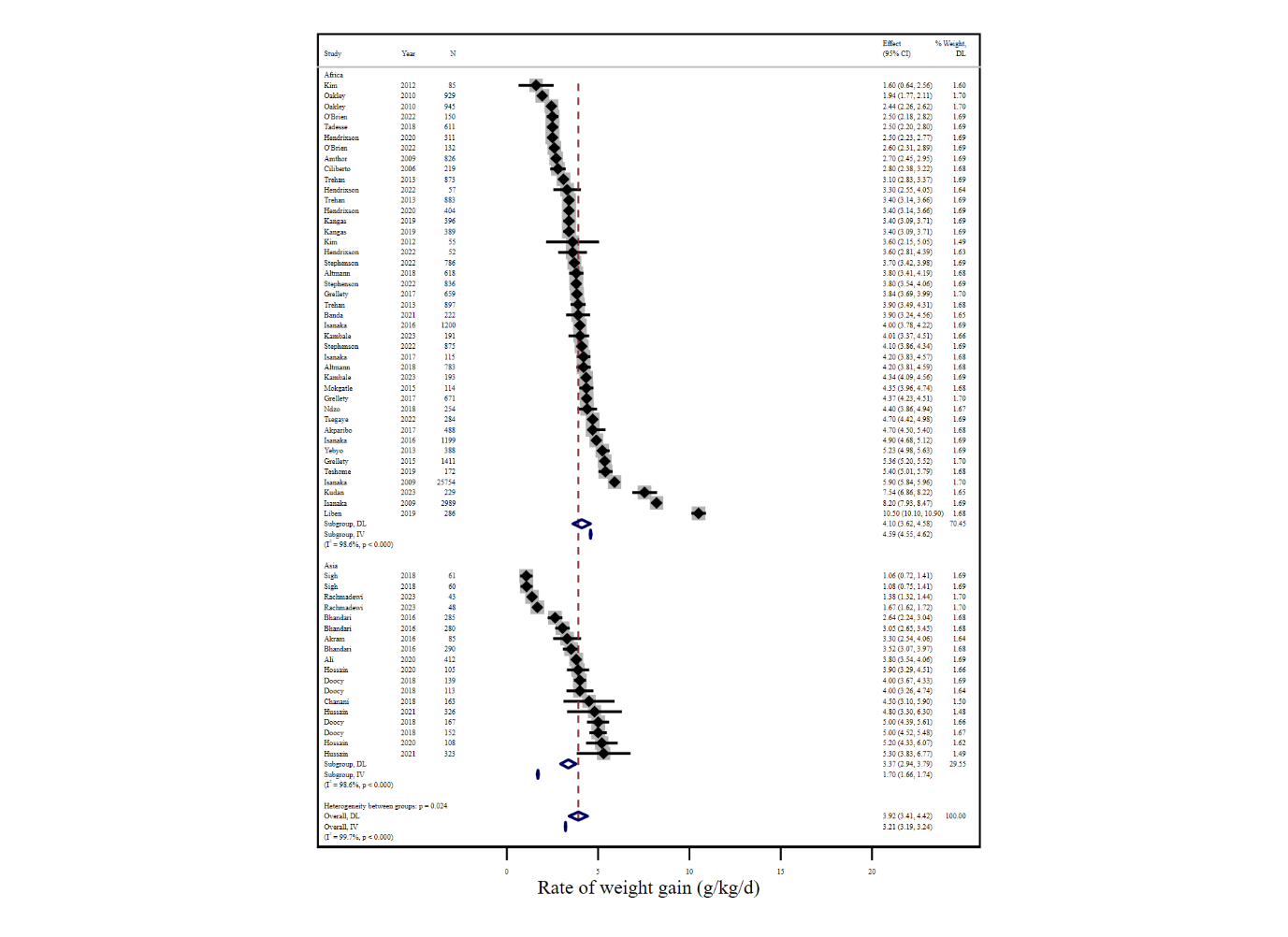


## Figure S4a. Weight gain (g/kg/d) among children with oedematous malnutrition (random and fixed effects models) (n=12 programmes).


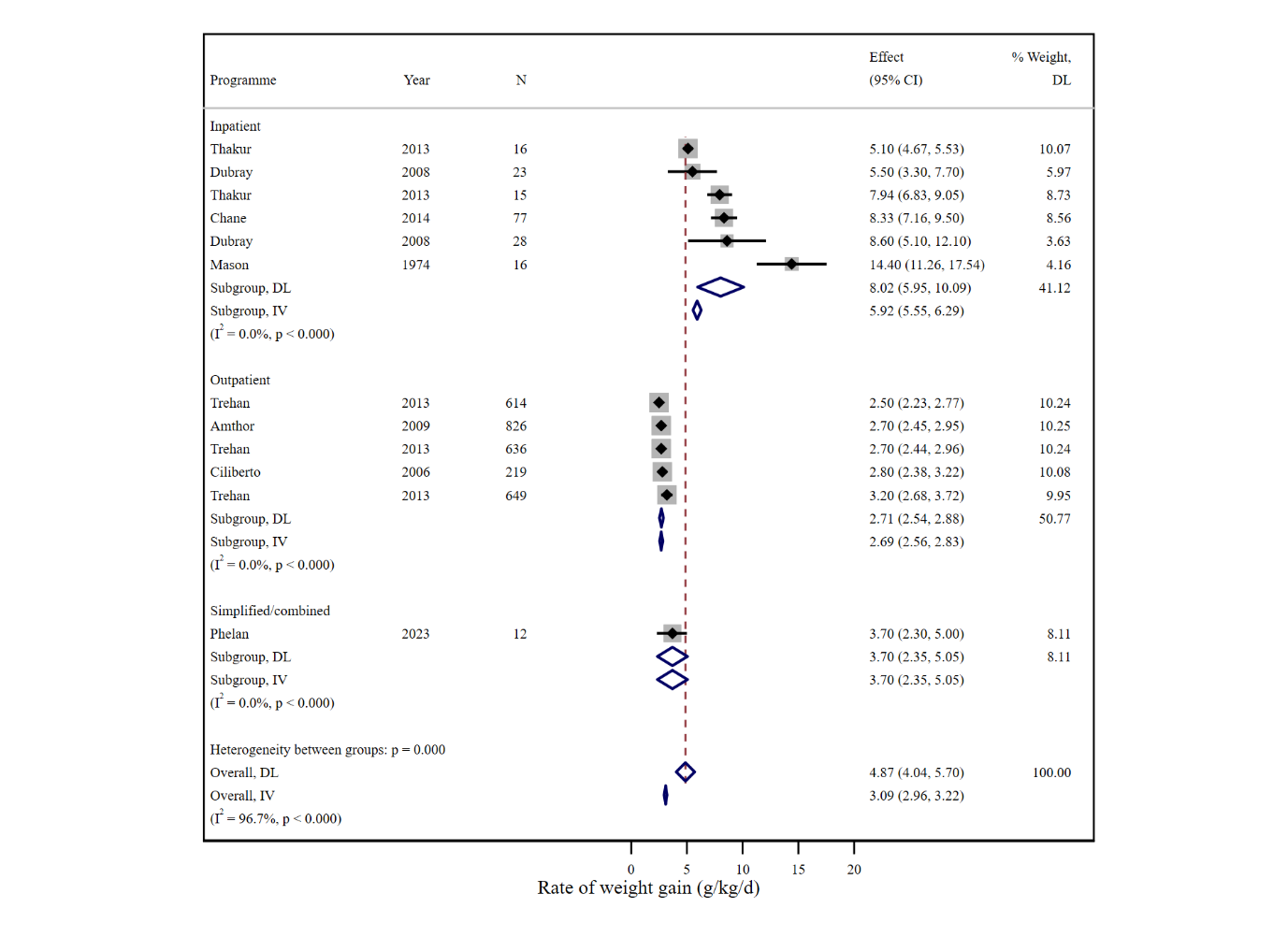


## Figure S4b. Weight gain (g/kg/d) among children with non-oedematous malnutrition (random and fixed effects models) (n=37 programmes).


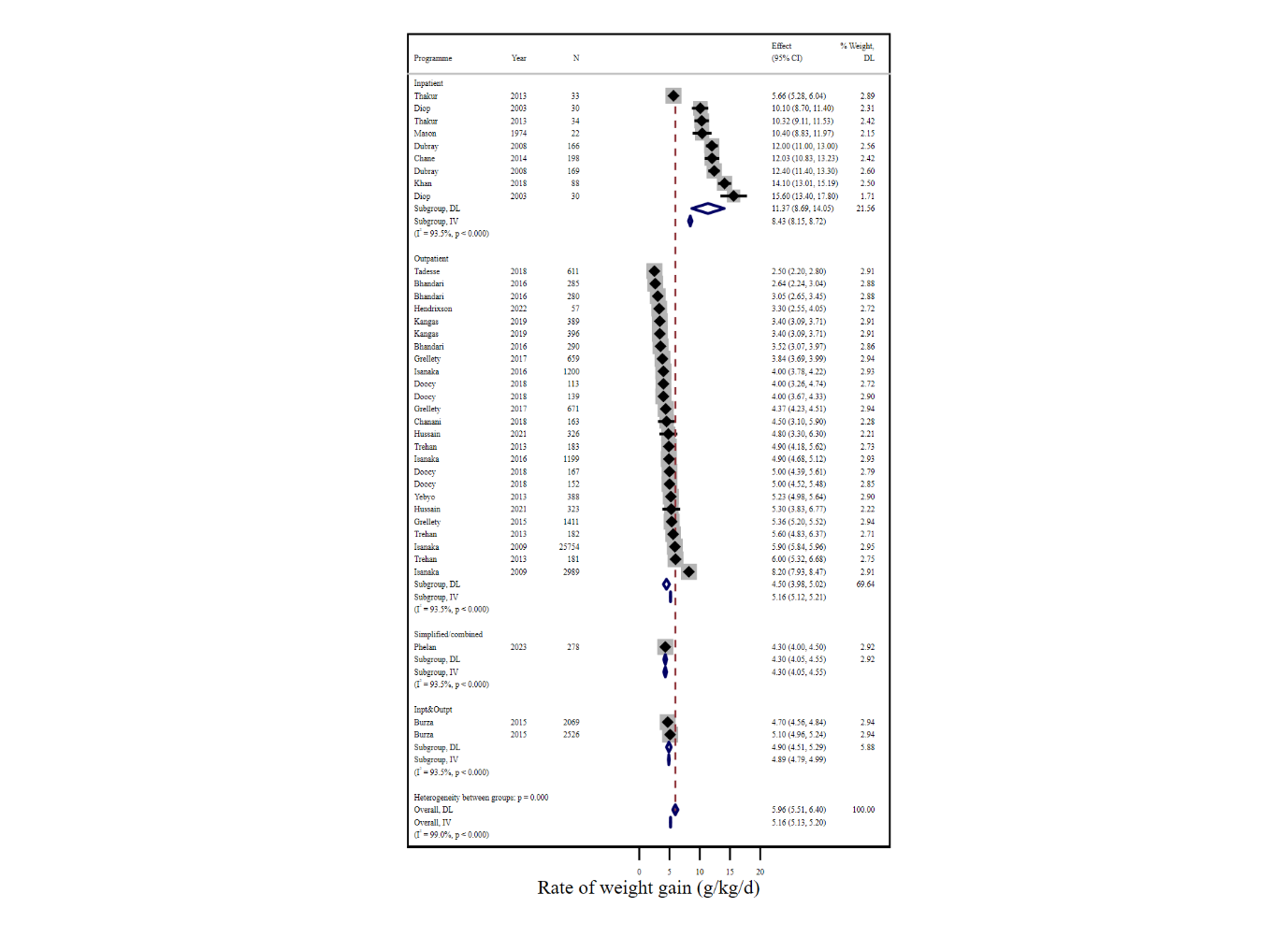


## Figure S5. Fixed-effects meta-analysis of mean length of stay (days) by inpatient (n=15), hybrid (n=8), and outpatient programmes (n=37).

**
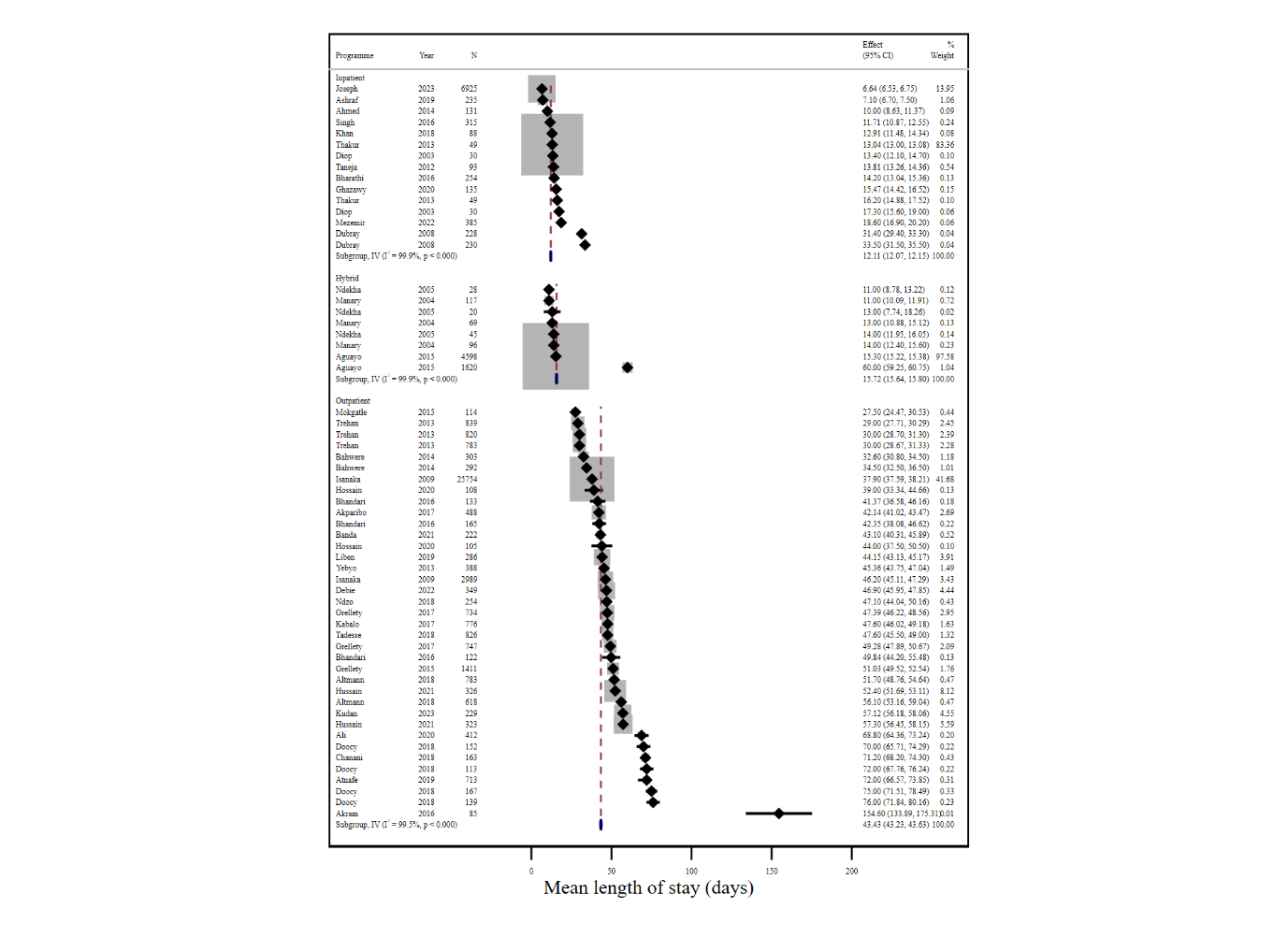
**

## Figure S6a. Random-effects meta-analysis of mean length of stay (days) by inpatient (n=11) and outpatient programmes (n=17), using estimates for all children in-programme.


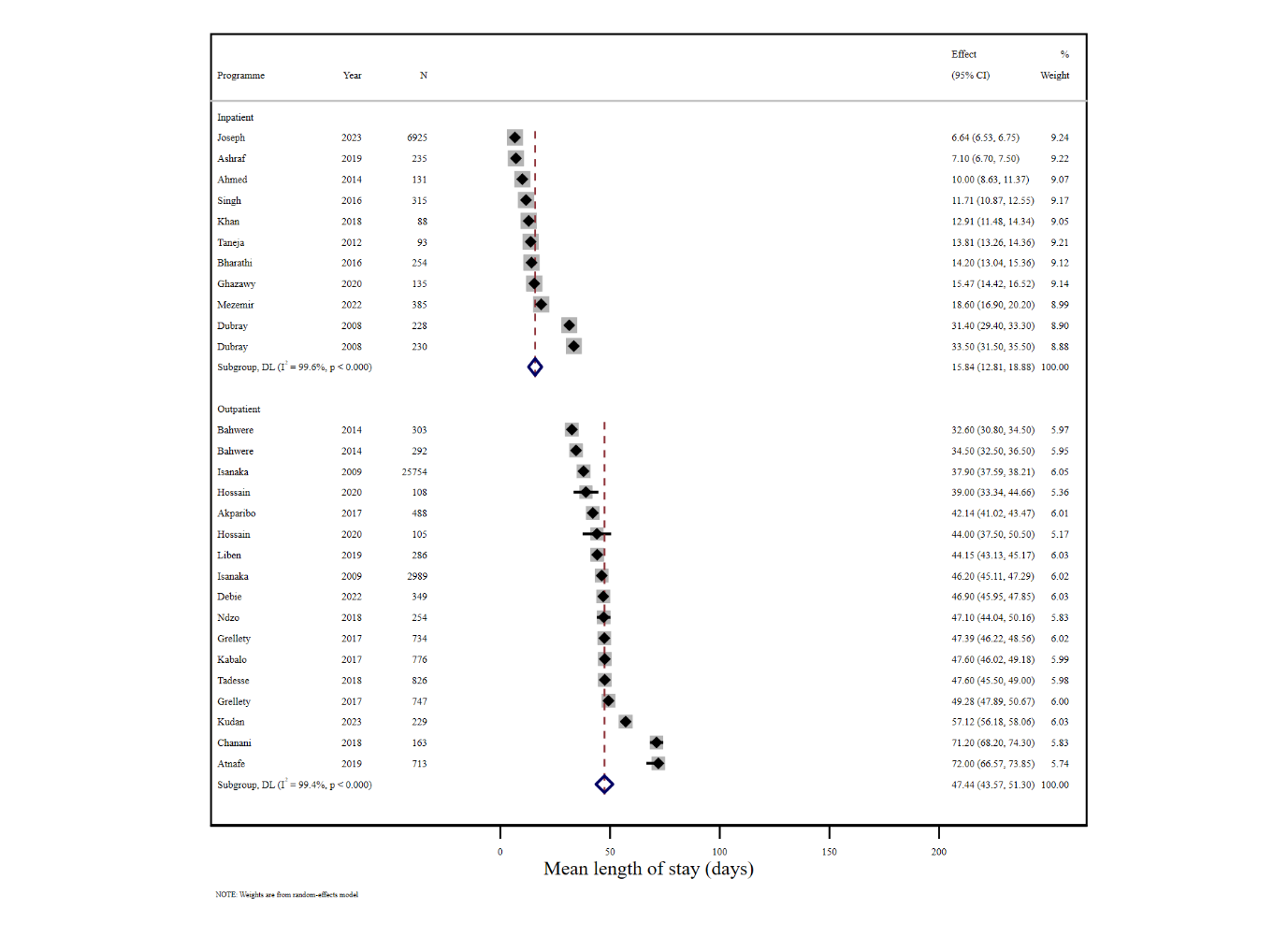


## Figure S6b. Random-effects meta-analysis of mean length of stay (days) by inpatient (n=5) and outpatient programmes (n=23), using estimates for only those children who recovered.


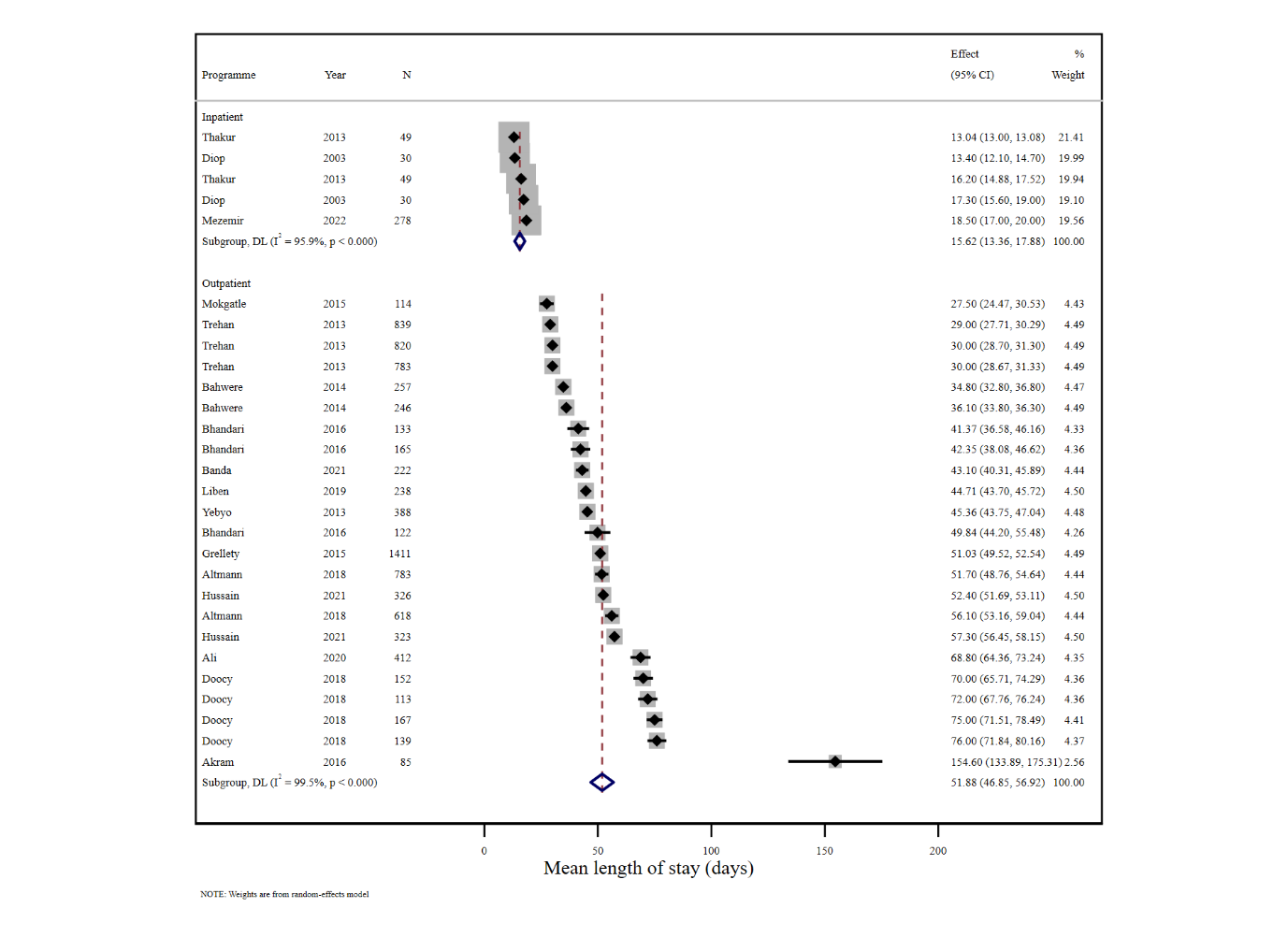


## Figure S6c. Fixed-effects meta-analysis of mean length of stay (days) by inpatient (n=11) and outpatient programmes (n=17), using estimates for all children in-programme.


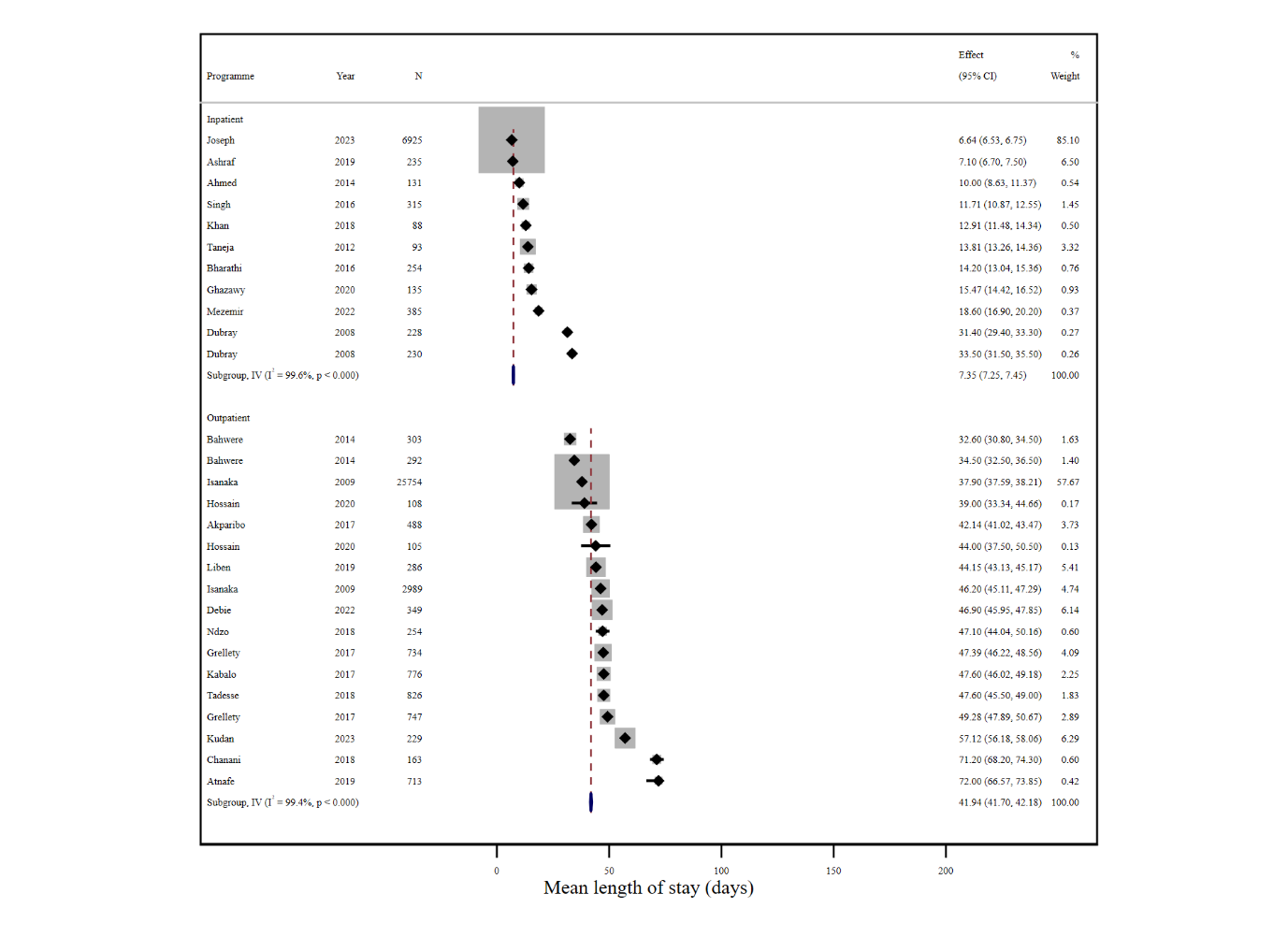


## Figure S6d. Fixed-effects meta-analysis of mean length of stay (days) by inpatient (n=5) and outpatient programmes (n=23), using estimates for only those children who recovered.


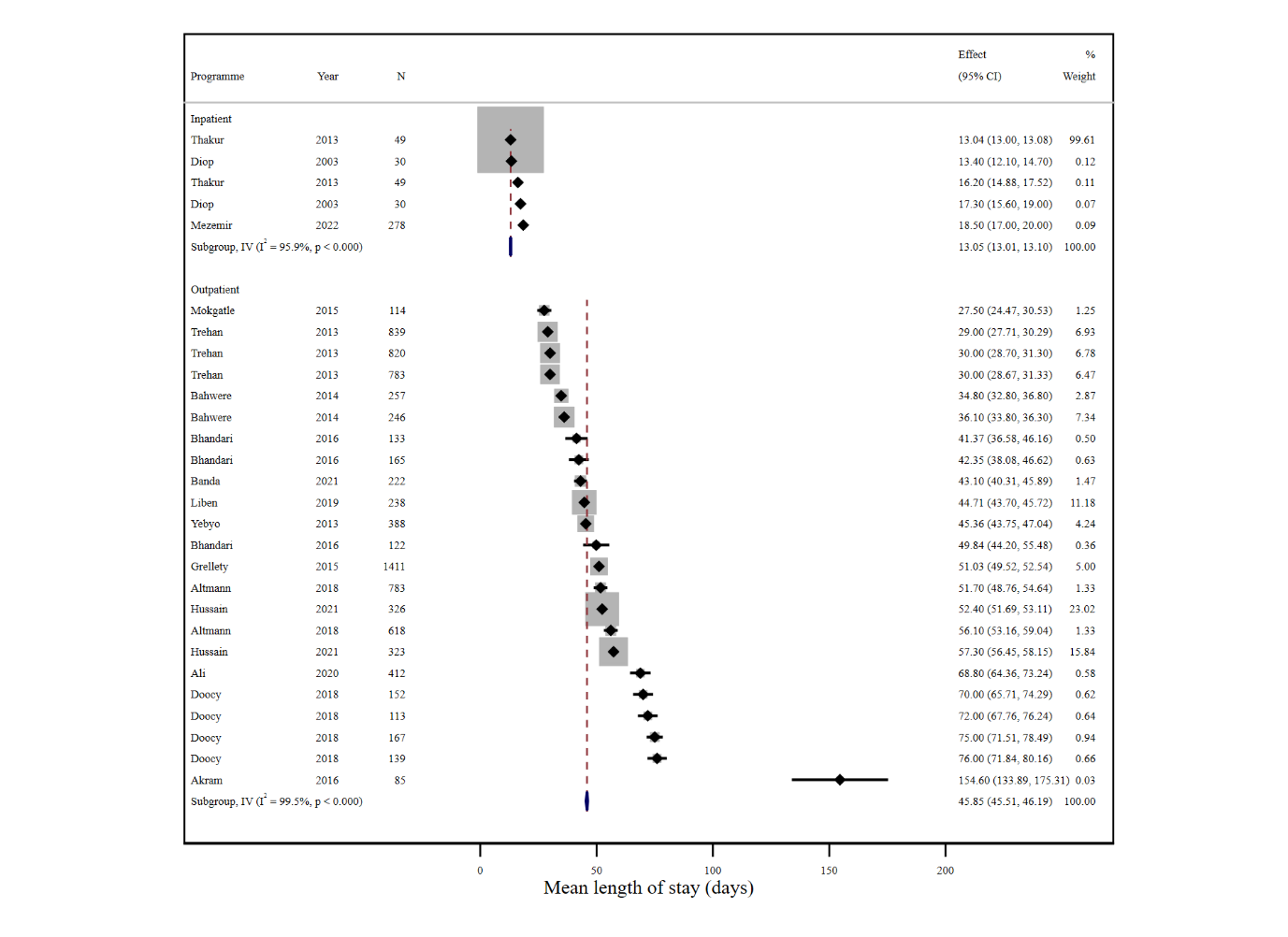


## Figure S7a. Mean length of stay (days) in inpatient programmes by region (random and fixed effects models) (n=15 programmes).


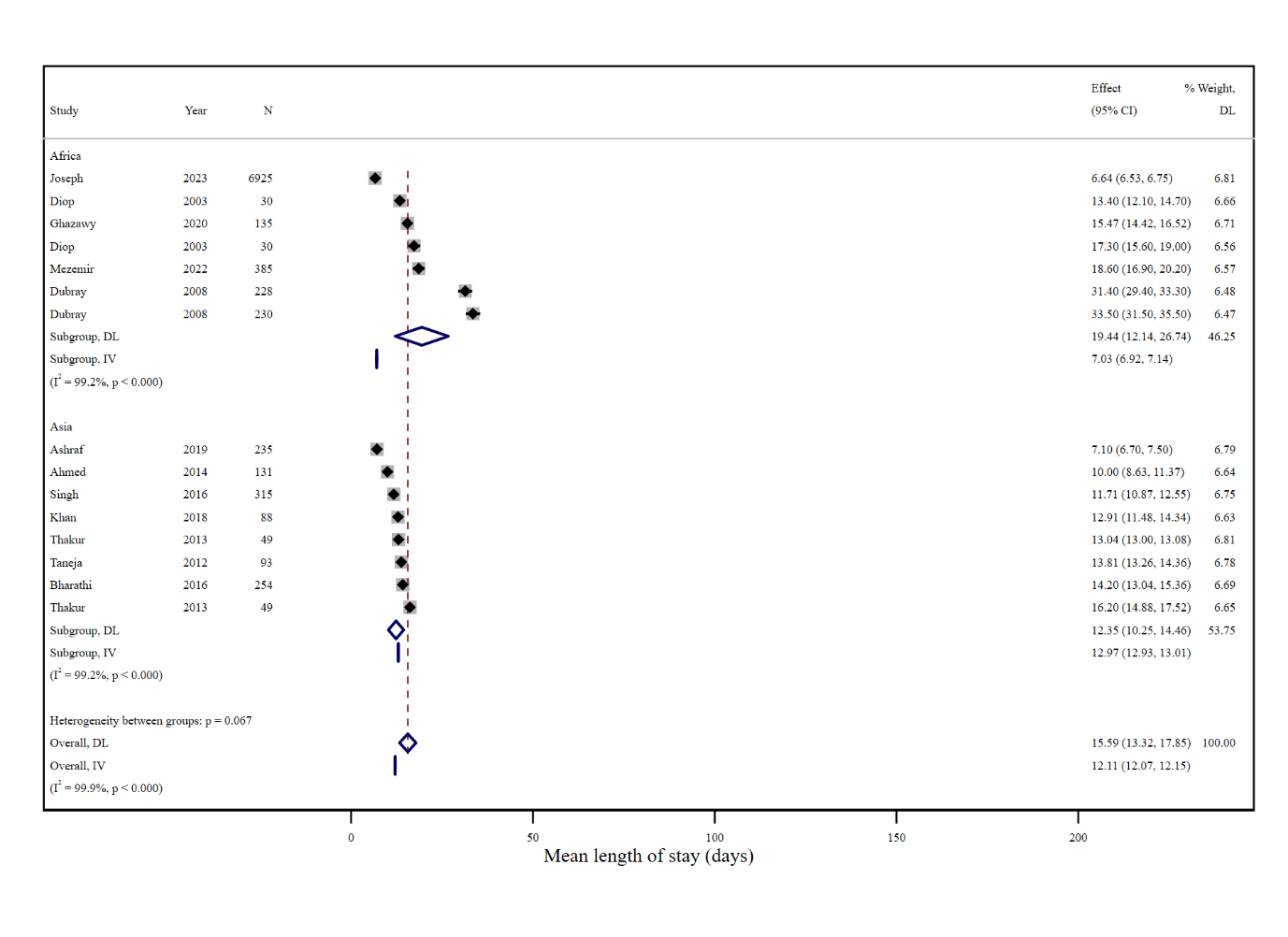


## Figure S7b. Mean length of stay (days) in outpatient programmes by region (random and fixed effects models) (n=37 programmes).


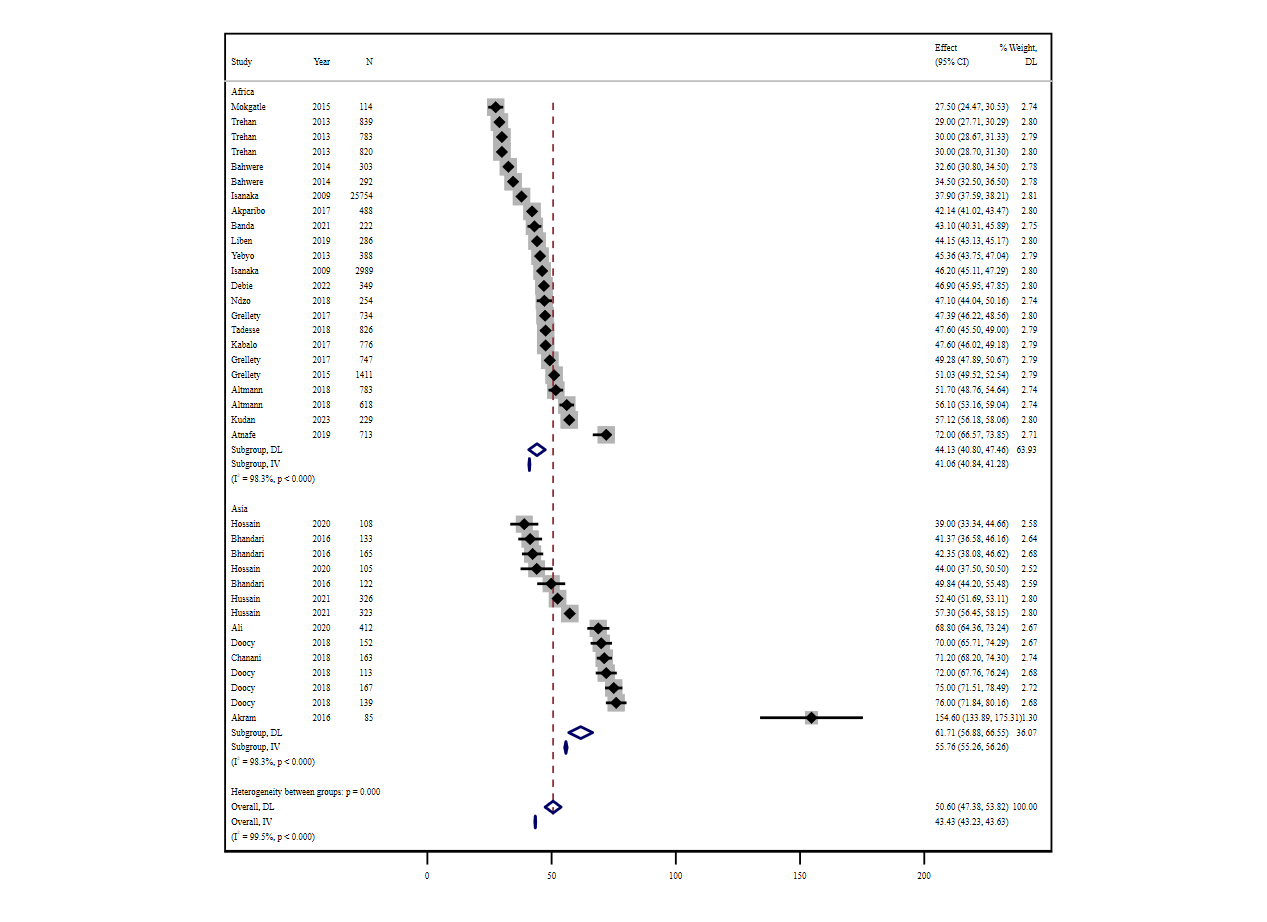


# References

1. Moramarco S, Amerio G, Ciarlantini C, et al. Community-based management of child malnutrition in Zambia: HIV/AIDS infection and other risk factors on child survival. *International Journal of Environmental Research and Public Health* 2016; **13**(7).
