# Supplementary material for: Weight gain among children under five with severe malnutrition in therapeutic feeding programmes: a systematic review and meta-analysis
Source: eClinicalMedicine. 2025 Feb 12;81:103083. doi: 10.1016/j.eclinm.2025.103083 (PMC11872456; doi:10.1016/j.eclinm.2025.103083)
Supplement: CHANGE Group Collaborators [file mmc2.docx]

| **First names** | **Surnames** |
| --- | --- |
| - Grace - Daniel - Thandile - Kenneth - Mubarek - Amir - Laurentya - Debbie - Kimberley - Elizabeth - Tim J. - Albert - Natasha - Amelia C. - Suvi T. - Gemechu - Asha - Charles - Marko | - O’Donovan - Allen - Nkosi-Gondwe - Anujuo - Abera - Kirolos - Olga - Thompson - McKenzie - Wimborne - Cole - Koulman - Lelijveld - Crampin - Kangas - Ameya - Badaloo - Opondo - Kerac |
